# Elovl7 sensitizes podocytes to ferroptosis in podocytopathy by elongating polyunsaturated fatty acids

**DOI:** 10.1038/s41419-025-08144-4

**Published:** 2025-11-24

**Authors:** Minchao Kang, Xiaojiang Zhan, Xinyu Huang, Yiting Zhao, Xiao Wang, Qiuyu Li, Jinjun Zhu, Fei Liu, Meihe Li, Linnan Bai, Jiejun Wen, Xinni Wang, Lei Zhou, Ruipeng Wei, Jianbo Qing, Ping Yan, Mingxi Lu, Jianhua Mao, Junnan Wu

**Affiliations:** 1 Department of Nephrology, Sir Run Run Shaw Hospital, Zhejiang University School of Medicine, Hangzhou, China; 2 Department of Nephrology, The First Affiliated Hospital, Jiangxi Medical College, Nanchang University, Key Laboratory of Urinary System Diseases of Jiangxi Province, Nanchang, China; 3 Department of Nephrology, The Children’s Hospital, Zhejiang University School of Medicine, National Clinical Research Center for Child Health, Hangzhou, China; 4 Department of Renal Transplantation, Nephropathy Hospital, The First Affiliated Hospital of Xi’an Jiaotong University, Xi’an, China; 5 Pediatric Translational Medicine Institute, Shanghai Children’s Medical Center, Shanghai Jiao Tong University School of Medicine, Shanghai, China

**Keywords:** Kidney diseases, Glomerular diseases

## Abstract

Podocytopathy is an emerging global health concern characterized by the injury of podocytes through various direct or indirect mechanisms. Recent research has highlighted a potential link between podocyte loss and various programmed cell death pathways, while the precise mechanisms of podocyte injury remain ambiguous. We conducted single-nucleus RNA sequencing (snRNA-seq) on kidney tissues from adriamycin-induced nephropathy (AN) mice (BALB/c, male) and renal biopsy samples from patients with different types of podocytopathy, such as focal segmental glomerulosclerosis (FSGS), minimal change disease (MCD) and obesity-related glomerulopathy (ORG). We found podocytes in diseased groups exhibited elevated ferroptosis scores based on the gene module score of programmed cell death pathways. Targeted lipidomics analysis revealed high phospholipids (PLs) levels containing long-chain polyunsaturated fatty acyl (LC-PUFA) tails. Metabolic pathway activity analysis indicated dysregulation of fatty acid elongation in podocytes of the AN group. We further reveal that the upregulation of Elovl7 in injured podocytes led to the accumulation of PLs with LC-PUFA tails, resulting in heightened sensitivity to ferroptosis. The results were confirmed by podocyte specific Elovl7 knockout mice and Elovl7 knockdown podocyte cell line. In conclusion, our study visualized injured podocytes and substantial podocyte loss from multiple podocytopathies. This phenomenon could potentially be attributed to the increased synthesis of LC-PUFAs facilitated by Elovl7, which leads to accumulation of intracellular lipid peroxidation and ultimately leading to ferroptosis.

## Introduction

Podocytopathy refers to a renal disorder with proteinuria or nephrotic syndrome resulting from podocytes damage, and its prevalence is increasing worldwide [1]. Podocytes, the visceral epithelial cells of the glomeruli, are the most important layer of the glomerular filtration membrane. Podocytes are extremely susceptible to damage due to their terminal differentiation and inability to proliferate. Any form of podocyte injury such as podocyte aplasia, phenotypic transformation, depletion and dedifferentiation can lead to different types of glomerular disease [2].

Several mechanisms of podocyte death have been identified, including apoptosis, necroptosis, ferroptosis, and pyroptosis in various kidney diseases [3]. Each type of cell death is distinguished by its unique morphological characteristics. Apoptosis, characterized as the predominant mode of podocyte death, is primarily defined by the proteolytic cascade initiated by caspases [4, 5]. Pyroptosis, an inflammatory form of cell death, is triggered by intracellular sensors like NLRP3, leading to the lytic demise of podocytes [6, 7]. Additionally, necroptosis, a genetically regulated form of necrotic cell death, contributes to the pathogenesis of FSGS when activated in podocytes [8].

Ferroptosis is a type of oxidative cell death caused by phospholipid peroxidation [9, 10]. Studies have reported the involvement of ferroptosis in kidney injury in STZ-induced type I diabetic mice and db/db mice [11, 12]. Nevertheless, research on podocyte ferroptosis in glomerular disease is limited, and concrete evidence of the contribution of ferroptosis to the development of glomerular disease is currently insufficient.

Bisallylic groups in polyunsaturated fatty acids (PUFAs) are known to be highly susceptible to peroxidation, so that determines its susceptibility to ferroptosis [13, 14]. Fatty acid elongases play a crucial role in various PUFA synthesis pathways. ELOVL5, responsible for elongating unsaturated fatty acids from C18:3n-6 to C20:3n-6 (a critical step in arachidonic acid (AA) biosynthesis), has been reported to promote ferroptosis [15, 16]. ELOVL7 is also a fatty acid elongase, it has been proved that it mainly elongases 18-carbon fatty acids regardless of the saturation [17, 18].

In this study, snRNA-seq was performed on kidney tissues from AN mice and renal biopsy samples from MCD, FSGS, and ORG patients to investigate the mechanisms underlying podocyte injury. The findings revealed an upregulation of LC-PUFAs synthesized by Elovl7 in AN mouse and podocytopathy patients. This elevated LC-PUFAs production led to the accumulation of intracellular lipid peroxidation, ultimately culminating in podocyte ferroptosis.

## Results

### SnRNA-seq identified a cluster of injured podocytes

**Fig. 1:**
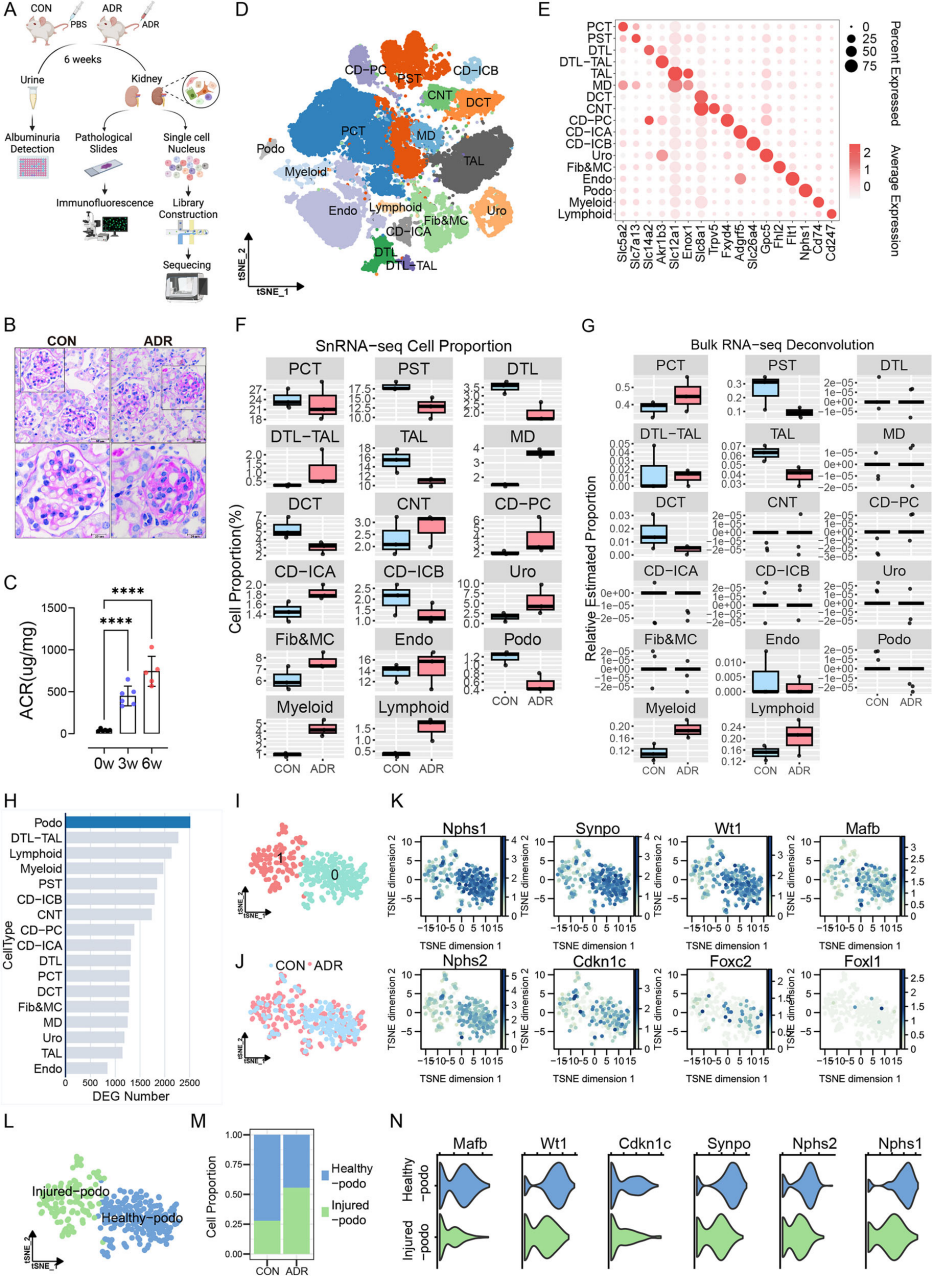
SnRNA-seq identified a cluster of injured podocytes. **A** Establishment process of ADR mouse model. Adriamycin was administered via the tail vein and both sides of kidney were harvested for measurements or snRNA-seq. **B** Representative PAS staining images of renal sections (*n* = 6). **C** Urinary albumin to creatinine ratio (ACR) at 0w, 3w and 6w after Adriamycin administration. Data are presented as mean ± SEM and statistical significance was assessed by one-way ANOVA followed by Tukey post-hoc test. ****p < 0.0001 vs 0w. **D** Umap plot showing 44,525 cells profiled from 6 mouse kidneys of healthy and diseased conditions (*n* = 3). **E** Dot plot showing expression of specific markers of each cell cluster. **F** Absolute proportion of each cell cluster in CON and ADR kidneys revealed by snRNA-seq (*n* = 3). **G** Cell proportions in CON and ADR kidneys quantified by bulk RNA-seq deconvolution (*n* = 3). **H** The number of DEGs of each cell type between CON and ADR group. **I** UMAP projection of all podocytes demonstrating original 2 subclusters. **J** UMAP projection of all podocytes grouped by CON and ADR. **K** Feature plots showing expression of specific podocyte markers. **L** UMAP projection of 2 subclusters annotated as Healthy-podo and Injured-podo. **M** Stacked bar chart showing subcluster cell proportion changes of podocytes in different groups. **N** Violin plot showing expression of specific podocyte markers in Healthy-podo and Injured-podo. PST proximal straight tubule, PCT proximal convoluted tubule, DTL descending limb of loop of Henle, TAL thick ascending limb of loop of Henle, MD macula densa, DCT distal convoluted tubule, CNT connecting tubule, CD-PC principal cell of collecting, CD-ICA type A intercalated cell of collecting duct, CD-ICB type B intercalated cell of collecting duct, Uro urothelium, Fib fibroblast, MC mesangial cell, Endo endothelial cell, Podo podocyte, Myeloid myeloid cell, Lymphoid lymphoid cell.

AN is a typical mouse model of human FSGS. We established the AN model on BALB/c mice to study the underlying mechanism of podocyte injury in podocytopathy (Fig. 1A). As expected, adriamycin (ADR) treatment caused severe glomerular sclerosis (Fig. 1B), elevated urinary albumin excretion (Fig. 1C), and reduced body weight (Fig. S1a) compared with the control group (CON). Using snRNA-seq, 44,525 cells from 6 kidney samples of mice (*n* = 3 CON and *n* = 3 ADR) were annotated into 17 cell types (Fig. 1D) based on their specifically highly expressed genes [19] (Fig. 1E, Fig. S1b) after filtering the number of features and percentage of mitochondrial genes in each cell (Fig. S1c). Cell proportions were largely changed between CON and ADR groups, podocyte numbers were obviously depleted in ADR kidney in snRNA-seq data and were confirmed in bulk RNA-seq (*n* = 3 CON and *n* = 3 ADR) deconvolution (Fig. 1F, G, Fig. S1d). Besides, proportions of proximal straight tubule (PST), descending limb of loop of Henle (DTL), Thick ascending limb of loop of Henle (TAL), distal convoluted tubule (DCT) were reduced, myeloid and lymphoid cells were increased in ADR kidney (Fig. 1F, G, Fig. S1d), indicating the presence of concurrent damage to other cell types and the inflammatory state of the kidneys. By counting the total number of differentially expressed genes (DEGs) between CON and ADR groups in each cell type, we found that podocytes had the largest number of DEGs (Fig. 1H), indicating ADR caused drastic changes in podocytes at the RNA level. It is worth noting that in bulk-RNAseq data, the DEGs between CON and ADR kidneys are barely expressed in podocytes but mostly expressed in PST and inflammatory cells (Fig. S2a), which is consistent with previous study of folic acid nephropathy [20]. This suggests that bulk RNA sequencing cannot capture the gene expression changes specific to podocytes due to the cellular heterogeneity in the kidney.

Next, A total of 352 podocytes were subjected to subgroup analysis. After subclustering podocytes, we identified two podocyte subsets: subset-0 and subset-1 (Fig. 1I). As shown in the T-distributed Stochastic Neighbor Embedding (TSNE) plot, podocytes of the CON group were mainly distributed in subset-0, while those of the ADR group mainly in subset-1 (Fig. 1J). The specific podocyte markers (*Nphs1, Nphs2, Synpo, Cdkn1c, Wt1, Foxc2, Mafb, Foxl1*) were expressed higher in subset-0 than subset-1 (Fig. 1K, Fig. S2b). Thus, we annotated subset-0 as Healthy-podo and subset-1 as Injured-podo (Fig. 1L). As expected, the proportion of Injured-podo increased in ADR group (Fig. 1M, Fig. S2c), and the expression levels of podocyte marker genes were lower in the Injured-podo than in the Healthy-podo (Fig. 1N). In summary, through snRNA-seq analysis, we identified a group of injured podocytes in ADR kidney.

### Injured podocytes were depleted in the form of ferroptosis

**Fig. 2:**
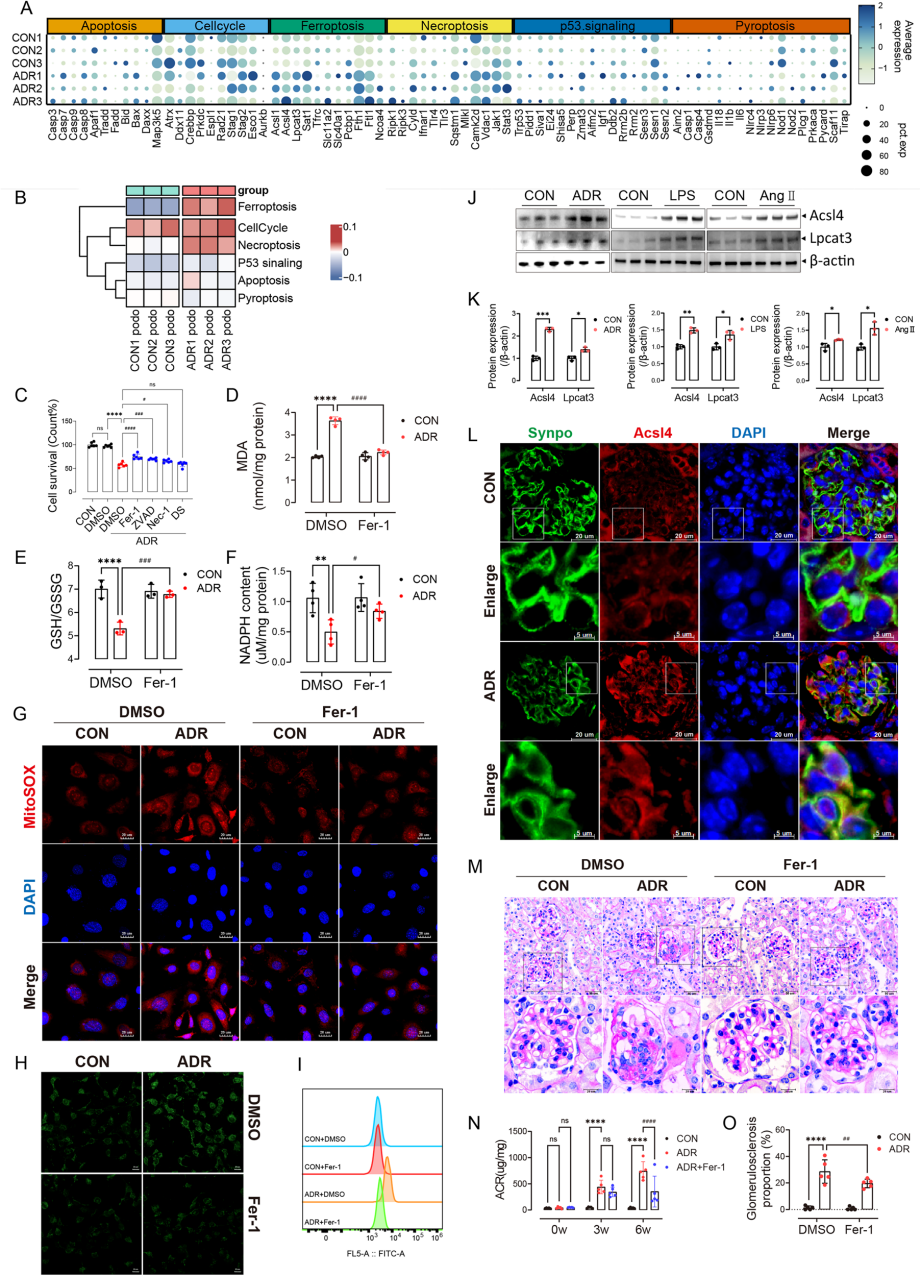
Injured podocytes were depleted in the form of ferroptosis. **A** Dot plot showing expression of genes involved in cell death pathways in snRNA-seq data. **B** Heat map showing means of gene module score of cell death pathways for podocytes of each sample. **C** Effect of different cell death inhibitors on cell viability after ADR treatment (*n* = 6). Data are presented as mean ± SEM and statistical significance was assessed by one-way ANOVA followed by Tukey post-hoc test. ****p < 0.0001 vs DMSO group; *#p* < 0.05, *###p* < 0.001, *####p* < 0.0001 vs “ADR + DMSO” group; ns, not significant. **D**–**F** Effects of ferroptosis inhibitors on the contents of MDA (*n* = 4), GSH (*n* = 3) and NADPH (*n* = 4). Data are presented as mean ± SEM and statistical significance was assessed by one-way ANOVA followed by Tukey post-hoc test. **p < 0.01, ****p < 0.0001 vs “CON + DMSO” group; *#p* < 0.05, *###p* < 0.001, *####p* < 0.0001 vs “ADR + DMSO” group. **G** Mitochondrial superoxide detected by MitoSOX in MPC5 cells under different conditions (*n* = 3). **H** Lipid peroxide stained by Liperfluo in MPC5 cells under different conditions (*n* = 3). **I** Liperfluo assessment elvaluated by flow cytometry. **J** Immunoblots of Acsl4, Lpcat3 in MPC5 cells treated with ADR, LPS or AngⅡ (*n* = 3). **K** Quantification of Acsl4, Lpcat3 normalized to β-actin for I (*n* = 3). Data are presented as mean ± SEM and statistical significance was assessed by two-tailed unpaired Student’s *t* test. *p < 0.05, **p < 0.01, ***p < 0.001 vs CON group. **L** Immunofluorescence of Acsl4 co-localized with Synpo (Podocyte-specific expressed protein) in CON or ADR-treated kidney sections (*n* = 6). **M** Representative PAS staining images of renal sections for different treatment of mice (*n* = 5). **N** ACR at 0w, 3w, 6w after adriamycin with or without Fer-1 treatment in different groups of mice (*n* = 5). Data are presented as mean ± SEM and statistical significance was assessed by one-way ANOVA followed by Tukey post-hoc test. ****p < 0.0001 vs CON group; ^*####*^*p* < 0.0001 vs ADR group; ns, not significant. **O** Glomerulosclerosis proportions were down regulated by Fer-1 in ADR mice (*n* = 5). Data are presented as mean ± SEM and statistical significance was assessed by one-way ANOVA followed by Tukey post-hoc test. ****p < 0.0001 vs “CON + DMSO” group; ^*##*^*p* < 0.01 vs “ADR + DMSO” group.

We further explored the mechanism of podocyte depletion in ADR mice by evaluating the programmed cell death pathways, including Apoptosis, Necroptosis, Pyroptosis, Cellcycle and P53 signaling pathways. We first calculated the gene module score of the programmed cell death gene sets (Table S1) for podocytes. Of all 6 cell death pathways, the Ferroptosis pathway exhibited significantly activated in ADR group, and podocytes in the ADR group expressed higher levels of genes associated with ferroptosis (Fig. 2A, B), especially *Acsl1, Acsl4, Lpcat3, Sat1, Fth1, Ftl1, Ncoa4* (Fig. S2d). ACSL4 is an enzyme that esterifies specific polyunsaturated fatty acids (PUFAs) into PUFA-CoA [21, 22], and LPCAT3 is responsible for incorporating PUFA-CoA into phospholipids and contributes to ferroptosis by triggering phospholipid peroxidation [23]. As note, among all cell types, *Acsl4* was most significantly upregulated in podocytes (Fig. S2e). Interestingly, necroptosis scores also exhibited higher levels in podocytes of ADR group to some extent, suggesting that necroptosis may also be involved in the podocyte depletion induced by ADR, which is consistent with previously reported studies [8, 24, 25].

Mouse Podocyte Clone-5 (MPC5) cells were then treated with ADR to establish podocyte injury model in vitro. We used a ferroptosis inhibitor ferrostatin-1 (Fer-1), an apoptosis inhibitor z-VAD-fmk (ZVAD), a necroptosis inhibitor necrostatin-1 (Nec-1) and a pyroptosis inhibitor disulfiram (DS) to further distinguish which type of programmed cell death participate in the podocyte death induced by ADR. Notebly, our data demonstrate that Fer-1, Z-VAD, and Nec-1 each partially restored cell viability (Fig. 2C), indicating the concurrent involvement of ferroptosis, apoptosis, and necroptosis in podocyte death. This conclusion was further supported by flow cytometry analysis showing Z-VAD’s specific rescue effect on ADR-induced apoptosis (Fig. S2f).

Ferroptosis is characterized by lipid peroxide accumulation [26, 27]. Next, we evaluated the levels of lipid peroxide terminal products such as MDA, as well as important antioxidants for counteracting ferroptosis, namely GSH and NADPH, to assess the extent of lipid peroxides in podocytes [28]. ADR increased the MDA levels, decreased the GSH/GSSG ratio, and reduced NADPH levels in ADR podocytes, which were partially reversed by Fer-1 (Fig. 2D–F). In addition, ADR elevated mitochondrial ROS and lipid peroxides in MPC5 cells as indicated by MitoSOX and Liperfluo staining respectively, and Fer-1 reversed this effect (Fig. 2G–I, Fig. S2g, h). The western blots showed that the protein levels of Acsl4 and Lpcat3 were increased in multiple podocyte injury models treated with ADR, lipopolysaccharide (LPS) or angiotensin II (AngII) (Fig. 2J, K). The expression of ferroptosis-related genes (*Acsl4*, *Lpcat3*, *Gpx4* and *Slc7a11*) in ADR podocytes were also proved by RT-qPCR (Fig. S2i). Immunofluorescence co-localization of Acsl4 and Synaptopodin (Synpo) further showed the increased Acsl4 expression in ADR mice podocytes (Fig. 2L, Fig. S2j, k).

Next, we confirmed the protective effect of the ferroptosis inhibitor on podocytes in vivo. Fer-1 was intraperitoneally injected every other day for 6 weeks after an intraperitoneal injection of ADR. Fer-1 effectively protected against glomerulosclerosis and albuminuria (Fig. 2M–O) induced by ADR administration. The body weights of mice were also partially recovered by Fer-1 compared with mice injected with ADR alone (Fig. S2l). In summary, these findings suggest that ferroptosis may represent a significant mechanism of podocyte death in glomerular disease.

### Lipid metabolism were disrupted in injured podocytes

**Fig. 3:**
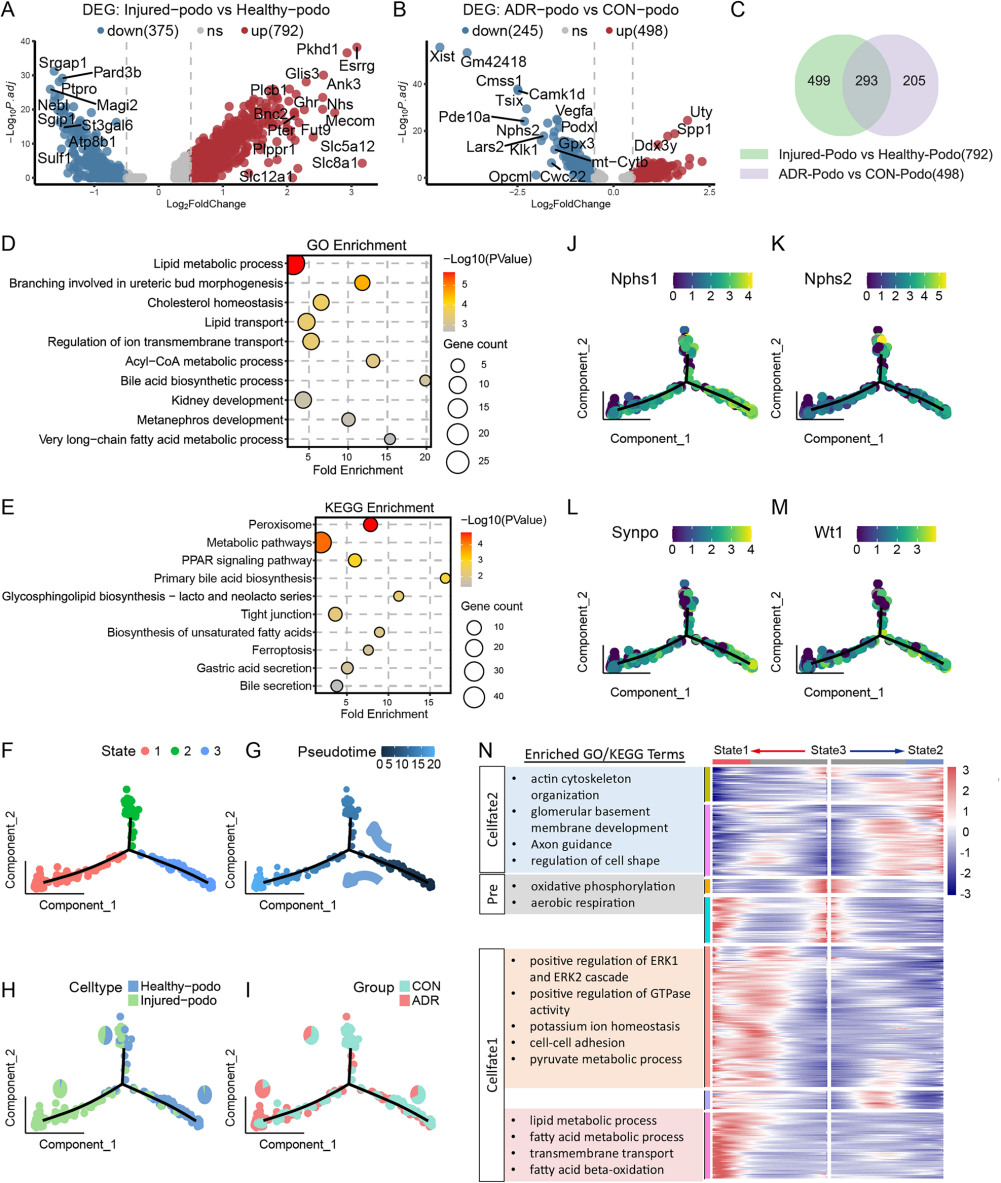
Lipid metabolism is disrupted in injured podocytes. **A** Volcano plot showing DEGs in comparisons between Injured-podo and Healthy-podo. **B** Volcano plot showing DEGs in comparisons between ADR-podo and CON-podo. **C** Venn diagram showing intersection genes of upregulated genes in Injured-podo and ADR-podo. **D** Bubble plot showing gene ontology (GO) enriched terms of the 293 intersection genes in (**C**). **E** Bubble plot of Kyoto Encyclopedia of Genes and Genomes (KEGG) enriched pathways of the 293 intersection genes in (**C**). **F** Pseudotime analysis showed three distinct states for all podocytes. **G** Pseudotime analysis showed two branches from one point for all podocytes. **H** Pseudotime trajectory of all podocytes colored by different clusters. **I** Pseudotime trajectory of all podocytes colored by different groups. **J**–**M** Expression of podocyte markers along pseudotime trajectory. **N** Enrichment terms of genes changed in expression along two branched trajectories.

We further explored the mechanism of podocyte ferroptosis. DEGs analysis of podocytes showed 1167 genes changed between two subclusters (Healthy-Podo and Injured-Podo) and 743 genes changed between different groups (CON-Podo and ADR-Podo) (Fig. 3A, B). We got 293 genes that both upregulated in Injured-podo and ADR-podo (Fig. 3C). GOBP and KEGG enrichment analysis of these genes revealed many terms involved in lipid metabolism such as *Lipid transport*, *Very long-chain fatty acid metabolic process*, *Peroxisome*, *PPAR signaling pathway* and *Biosynthesis of unsaturated fatty acids* (Fig. 3D, E), suggesting dysregulated lipid metabolism in injured podocytes. Ferroptosis pathway was also enriched in the KEGG enrichment analysis (Fig. 3E). We next took advantage of trajectory analysis for all podocytes using monocle2 package and identified two distinct trajectories starting from State 3 (Fig. 3F, G), which is occupied by most of the podocytes from Healthy-podo or CON group (Fig. 3H, I) and expresses higher levels of podocyte marker genes (Fig. 3J–M). These cells gradually branched over pseudotime and some of them became almost Injured-podo at the State1 (Fig. 3H), which contains most of the podocytes from ADR group (Fig. 3I) and expresses lower levels of podocyte marker genes (Fig. 3J–M). Next, we pulled genes whose expression changed along the two trajectories: genes ascending expressed from State3 to State 1 (Cellfate 1) were enriched in lipid metabolic process, which was in accord with the DEGs enrichment results above; genes ascending expressed from State3 to State 2 (Cellfate 2) were enriched in terms related to podocyte skeleton development (Fig. 3N), indicating a normal differentiation process of podocytes. Genes involvled in cytoskeleton development are also downregulated in State 1, an early sign of podocyte injury [29].

There is extensive evidence linking lipid metabolism disturbances to ferroptosis [10, 13, 14, 30]. Moreover, in a kidney snRNAseq data of Pdss2^kd/kd^ [KDKD] (kidney disease) mice with CoQ deficiency resulting in inadequate protection against PUFAs peroxidation [31], ferroptosis score in KDKD podocytes was also upregulated compared with CTRL podocytes (Fig. S2m). This suggests that the abnormal lipid metabolism in podocytes promote the occurrence of ferroptosis.

In summary, these results indicate that ADR-treated podocytes exhibit significant disturbances in lipid metabolism and may potentially promote podocyte ferroptosis.

### Phospholipids with long-chain polyunsaturated fatty acyl tails increased in injured podocytes

**Fig. 4:**
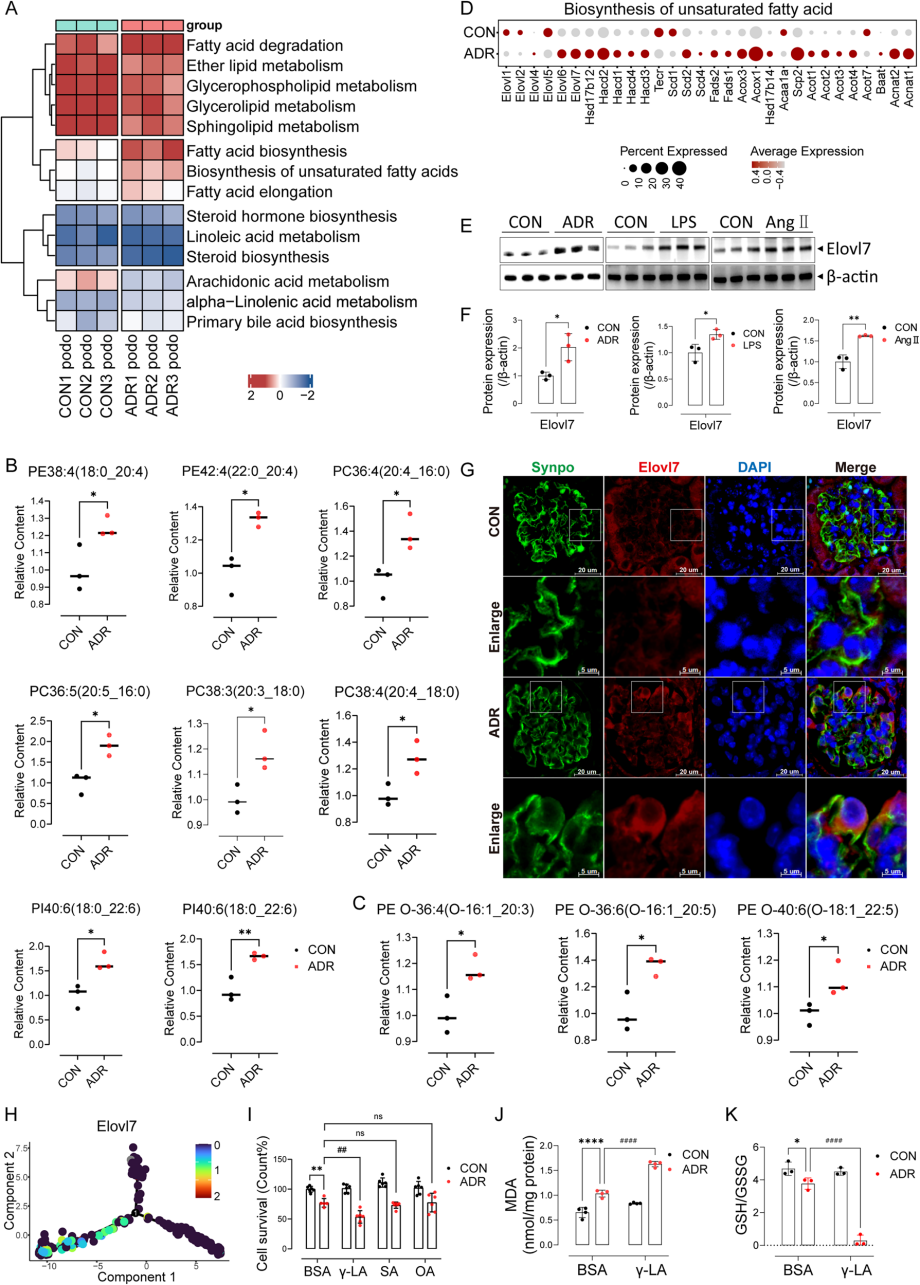
Phospholipids with long-chain polyunsaturated fatty acid tails increased in injured podocytes. **A** Gene module scores of lipid metabolism pathways for podocytes from snRNA-seq data. **B** Relative contents of representative PLs with LC-PUFA tails in CON or ADR-treated cells (*n* = 3). **C** Relative contents of representative oxidative PLs in CON or ADR-treated cells (*n* = 3). Data are presented as mean ± SEM and statistical significance was assessed by two-tailed unpaired Student’s t-test. *p < 0.05 vs CON group. **D** Dot plot showing expression of genes involved in the *Biosynthesis of unsaturated fatty acid* pathway in podocytes of snRNA-seq data. **E** Immunoblots of Elovl7 in MPC5 cells treated by ADR, LPS or AngⅡ (*n* = 3). **F** Quantification of Elovl7 blots normalized to β-actin for E (*n* = 3). Data are presented as mean ± SEM and statistical significance was assessed by two-tailed unpaired Student’s *t* test. *p < 0.05, **p < 0.01 vs CON group. **G** Immunofluorescence of Elovl7 co-localized with Synpo in CON or ADR-treated kidney sections (*n* = 6). **H** Expression of Elovl7 along the pseudotime trajectory of podocytes. **I** Cell survival rate of ADR-treated MPC5 cells with γ-LA, SA or OA supplementation (*n* = 6). **J** Content of MDA in CON or ADR cells with γ-LA stimulation (*n* = 4). **K** GSH/GSSG were detected in CON or ADR cells with γ-LA stimulation (*n* = 3). Data are presented as mean ± SEM and statistical significance was assessed by one-way ANOVA followed by Tukey post-hoc test. *p < 0.05, **p < 0.01 ****p < 0.0001 vs “CON + BSA” group; ^*##*^*p* < 0.01, ^*####*^*p* < 0.0001 vs “ADR + BSA” group; ns not significant.

To investigate which lipid metabolic process contributes to podocyte injury and death in ADR kidney, we further assessed the activity of lipid metabolism pathways activity for podocytes in snRNA-seq data. We found three pathway scores (*Fatty acid biosynthesis*, *Biosynthesis of unsaturated fatty acids* and *Fatty acid elongation*) were elevated in ADR podocytes compared with CON group (Fig. 4A).

**Fig. 5:**
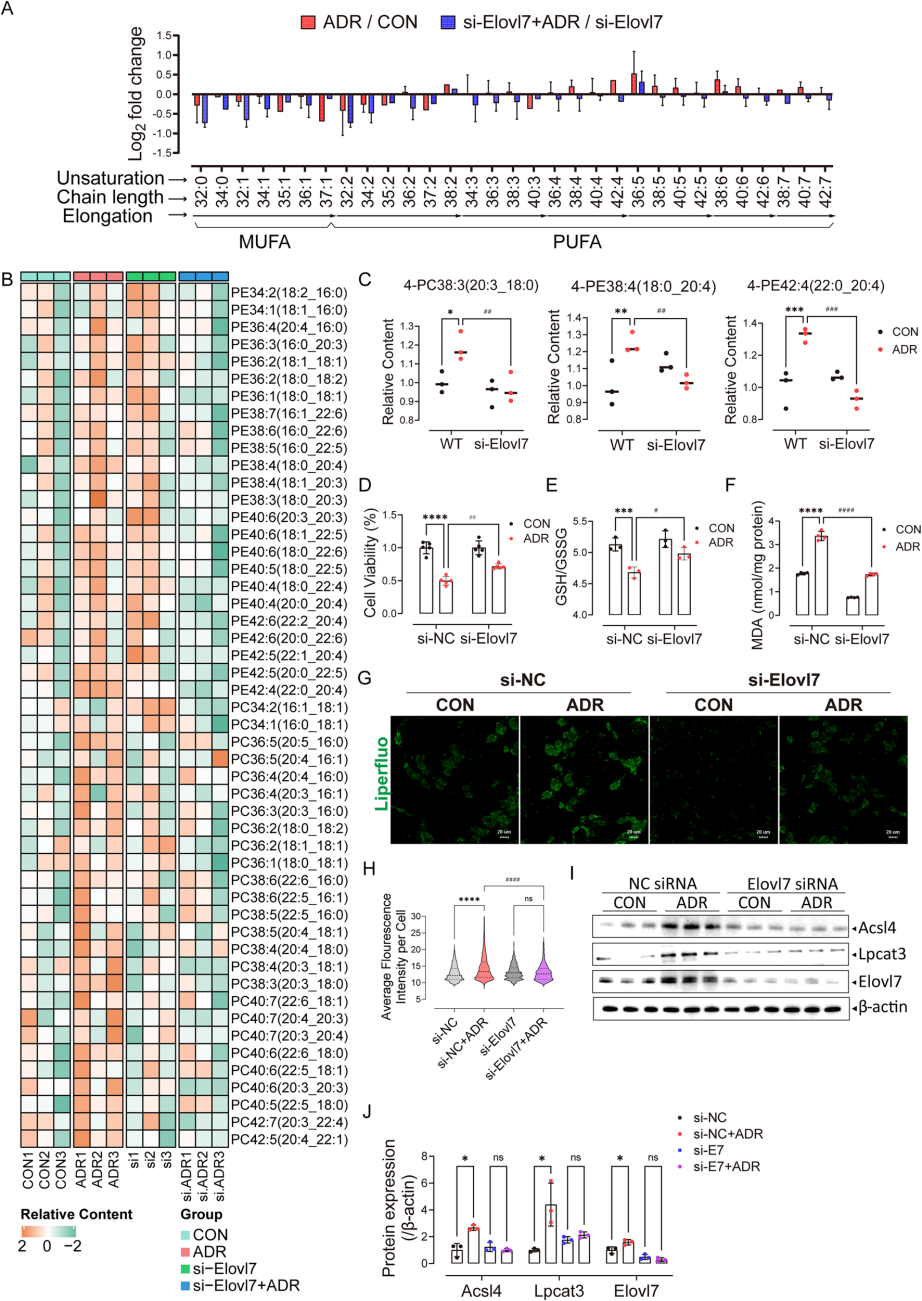
Elovl7 knockdown mitigated the elevation of LC-PUFA-PLs in injured podocytes and reduced podocyte ferroptosis. **A** Phospholipid abundance in ADR-treated cells versus CON cells along with the elongation of acyl chain length and the ascending of unsaturation of PL tails. **B** Heatmap showing relative contents of PE and PC with tails of different length and saturation in wide-type or si-Elovl7 cells with or without ADR treatment. **C** Relative contents of representative PLs with LC-PUFA tails in wide-type or si-Elovl7 cells with or without ADR treatment (*n* = 3). Data are presented as mean ± SEM and statistical significance was assessed by one-way ANOVA followed by Tukey post-hoc test. *p < 0.05, **p < 0.01, ***p < 0.001 vs WT group; ^*##*^*p* < 0.01, ^*###*^*p* < 0.001 vs “si-NC + ADR” group. **D** Cell survival rate of cells treated with si-NC or si-Elovl7 after ADR stimulation (*n* = 5). **E** GSH/GSSG were detected in cells treated with si-NC or si-Elovl7 after ADR stimulation (*n* = 3). **F** Content of MDA in cells treated with si-NC or si-Elovl7 after ADR stimulation (*n* = 4). Data are presented as mean ± SEM and statistical significance was assessed by one-way ANOVA followed by Tukey post-hoc test. ***p < 0.001, ****p < 0.0001 vs “si-NC” group; ^*#*^*p* < 0.05, ^*##*^*p* < 0.01, ^*####*^*p* < 0.0001 vs “si-NC + ADR” group. **G** Lipid peroxide in cells treated with si-NC or si-Elovl7 after ADR stimulation stained by Liperfluo (*n* = 3). **H** Quantification of Liperfluo intensity in podocytes treated in different condition. Data are presented as mean ± SEM and statistical significance was assessed by one-way ANOVA followed by Tukey post-hoc test. ****p < 0.0001 vs “si-NC” group; ^*####*^*p* < 0.0001 vs “si-NC + ADR” group. **I** Immunoblots of Acsl4, Lpcat3 and Elovl7 in cells treated with si-NC or si-Elovl7 after ADR stimulation (*n* = 3). **J** Quantification of Acsl4, Lpcat3 and Elovl7 blots normalized to β-actin in si-NC or si-Elovl7 cells after ADR treatment (*n* = 3). Data are presented as mean ± SEM and statistical significance was assessed by one-way ANOVA followed by Tukey post-hoc test. *p < 0.05 vs si-NC group; ns, not significant.

Since highly oxidizable PUFAs incorporated in phospholipids (PLs) drive cells to ferroptosis [32], we performed targeted lipidomics of podocytes with ADR treatment. Orthogonal partial least squares discriminant analysis (OPLS-DA) clearly differentiated ADR and CON samples (Fig. S3a). Among four major phospholipids that make up the cell membrane (phosphatidylethanolamine (PE), phosphatidylcholine (PC), phosphatidylinositol (PI), phosphatidic acid (PA)), most phospholipids with PUFA tails content increased (Fig. S3b, c), especially PE and PC with LC-PUFA tail such as arachidonic acid (AA, C20:4), eicosapentaenoic acid (EPA, C20:5), docosahexaenoic acid (DHA, C22:6) (Fig. 4B), showing a positive log2FC value of ADR/CON (Fig. 5A). The level of oxidized PLs was also increased in ADR samples (Fig. 4C, Fig. S3d). Interestingly, PUFAs promote ferroptosis, whereas MUFAs limit ferroptosis due to the different structure of PUFA and MUFA.

Our lipidomics data revealed that while SFA and MUFA containing phospholipids (e.g., PE (18:1_18:1), PE (18:0_18:1)) were elevated (>1-fold, Fig. S3e), PUFA phospholipids showed more pronounced accumulation, with most species significantly increased (>1-fold, Fig. S3f). The SFA-MUFA/PUFA ratio shifted from 1.06 (>1, SFA/MUFA-dominant) in controls (CON) to 0.88 (<1, PUFA-dominant) in the ADR group, reflecting a transition to PUFA predominance. Furthermore, the relative SFA-MUFA/PUFA ratio (ADR vs. CON) was 0.82 (<1), confirming that PUFA phospholipids increased disproportionately compared to SFA/MUFA species.

We then checked gene expression levels involved in PUFA synthesis in podocytes of snRNA-seq data (Fig. 4D, Fig. S4a, b). Several genes involved in *Biosynthesis of unsaturated fatty acids* were upregulated in ADR podocytes, including two desaturases *Fads1* and *Fads2* that have been reported to synthesize LC-PUFAs and be associated with a variety of diseases [33–35]. Although a key enzyme *Elovl5* promoting ferroptosis in gastric cancer cells [16], was no increase in expression, another elongase *Elovl7* was increased (Fig. 4D), which catalyzes the rate-limiting step in the synthesis of very long-chain fatty acids and exhibits the highest activity toward 18-carbon fatty acids [17, 36]. The upregulated protein expression of Elovl7 in ADR, LPS and AngII treated podocytes was verified by western blotting in vitro (Fig. 4E, F) and immunofluorescence co-localization with Synpo in vivo (Fig. 4G, Fig. S4c, d). Ascending expression of *Elovl7* was also observed along the trajectory of podocyte damage (Fig. 4H). The module scores of *Ferroptosis*, *Fatty acid elongation* and *Biosynthesis of unsaturated fatty acids* along the podocytes trajectory also showed higher levels at the Injured-podo state (Fig. S4e).

While snRNA-seq detected elevated Elovl6 expression, its protein levels remained unchanged (Fig. S4f, g), consistent with its known preference for SFA/MUFA elongation (e.g., C16:0/1) [37]. Systematic evaluation of other Elovls (Fig. S4h) revealed: Elovl1 (responsible for C24 sphingolipids) and Elovl2 (testis-specific C24-C30 PUFA) did not match our lipid profile; Elovl4-upregulated VLC-FAs (>C24) were lipidomically undetectable; and Elovl5’s modest increase was statistically insignificant. These data collectively implicate Elovl7 as the primary driver of PUFA phospholipid accumulation due to its unique PUFA elongation specificity.

Fer-1 reacts with peroxide free radicals in cells and blocks the amplification process of chain reaction in lipid peroxidation, thereby inhibiting ferroptosis [38], so LC-PUFA-PLs were not significantly reduced after Fer-1 treatment in targeted lipidomics (Fig. S4i).

In order to determine which substrate that Elovl7 elongated, thus contributing to ferroptosis, we proceeded by incubating three fatty acid substrates of Elovl7 with podocytes following ADR stimulation: Gamma linolenic acid (γ-LA, containing three unsaturated bonds), oleic acid (OA, monounsaturated fatty acids) and stearic acid (SA, saturated fatty acids) (Fig. S5a). Supplementation with γ-LA significantly exacerbated the ADR-induced reduction in cell viability, increase in MDA content, and decrease in GSH/GSSG levels (Fig. 4I–K). Additionally, it exacerbated changes in the expression of genes involved in ferroptosis (*Alox15*, *Ptgs2*, *Gpx4*, *Slc7a11*), triacylglycerol synthesis (*Dgat1*, *Dgat2*, *Plin2*) and de novo synthesis (*Fasn*) (Fig. S5b). Lipidomics also confirmed that γ-LA aggravated the elevation of LC-PUFA-PLs (Fig. S5c), and Fer-1 reduced the increase of MDA induced by γ-LA (Fig. S5d).

We next conducted isotope tracing analysis using ^13^C18-linoleic acid to verify whether Elovl7 indeed elongates C18 unsaturated fatty acids. It can be observed that ^13^C18:2, ^13^C20:3 and ^13^C20:4 fatty acids were all elevated in ADR stimulated cells (Fig. S5e–g). This indicates that the flux from ¹³C18-linoleic acid to C20:3 was enhanced in the ADR group.

Hence, we postulated that the elongation of unsaturated fatty acids by Elovl7 could represent a crucial step in the synthesis pathway of LC-PUFAs, resulting in the augmentation of phospholipids containing LC-PUFA tails and rendering podocytes more susceptible to ferroptosis.

### Elovl7 knockdown mitigated the elevation of LC-PUFA-PLs in injured podocytes and reduced podocyte ferroptosis

To elucidate the critical function of Elovl7, we used small interfering RNA (siRNA) to knock down Elovl7 in podocytes (Fig. S6a, b). In the OPLS-DA plot of targeted lipidomics, Elovl7-knockdown cells were clearly separated from wild-type cells treated with ADR (Fig. S6c). Elovl7 knockdown limited the elevation and oxidation of the LC-PUFA-PLs after ADR stimulation, especially LC-PUFA-PE and LC-PUFA-PC (Fig. 5B, C, Fig. S5d, e), suggesting Elovl7 is a key enzyme to synthesis LC-PUFAs. In addition, interference with Elovl7 blocked the reduction in cell viability induced by ADR to some extent (Fig. 5D), alleviated the decline in GSH/GSSH levels (Fig. 5E) and abolished the lipid peroxides increase (Fig. 5F–H). The two enzymes (Acsl4, Lpcat3) important for synthesizing and remodeling membrane lipids were also downregulated after Elovl7 knockdown (Fig. 5I, J). Acsl4 introduces long-chain polyunsaturated ω6 fatty acids into cell membrane phospholipids [22, 39]. Our data suggest that ADR-induced elevation of LC-PUFAs (mediated by Elovl7) upregulates downstream enzymes Acsl4 and Lpcat3 via a feedforward mechanism. Notably, Elovl7 knockdown abolished this effect, as evidenced by reduced upstream LC-PUFA substrates and abrogation of Acsl4/Lpcat3 induction. Moreover, Elovl7 knockdown affected the expression of genes related to ferroptosis (*Alox15*, *Lpcat3*, *Gpx4*, *Slc7a11*), fatty acid desaturation (*Fads1*), triacylglycerol synthesis (*Dgat2*, *Plin2*) and de novo synthesis (*Fasn*) (Fig. S6f). We hypothesized that Elovl7 promotes ferroptosis by synthesizing LC-PUFA.

**Fig. 6:**
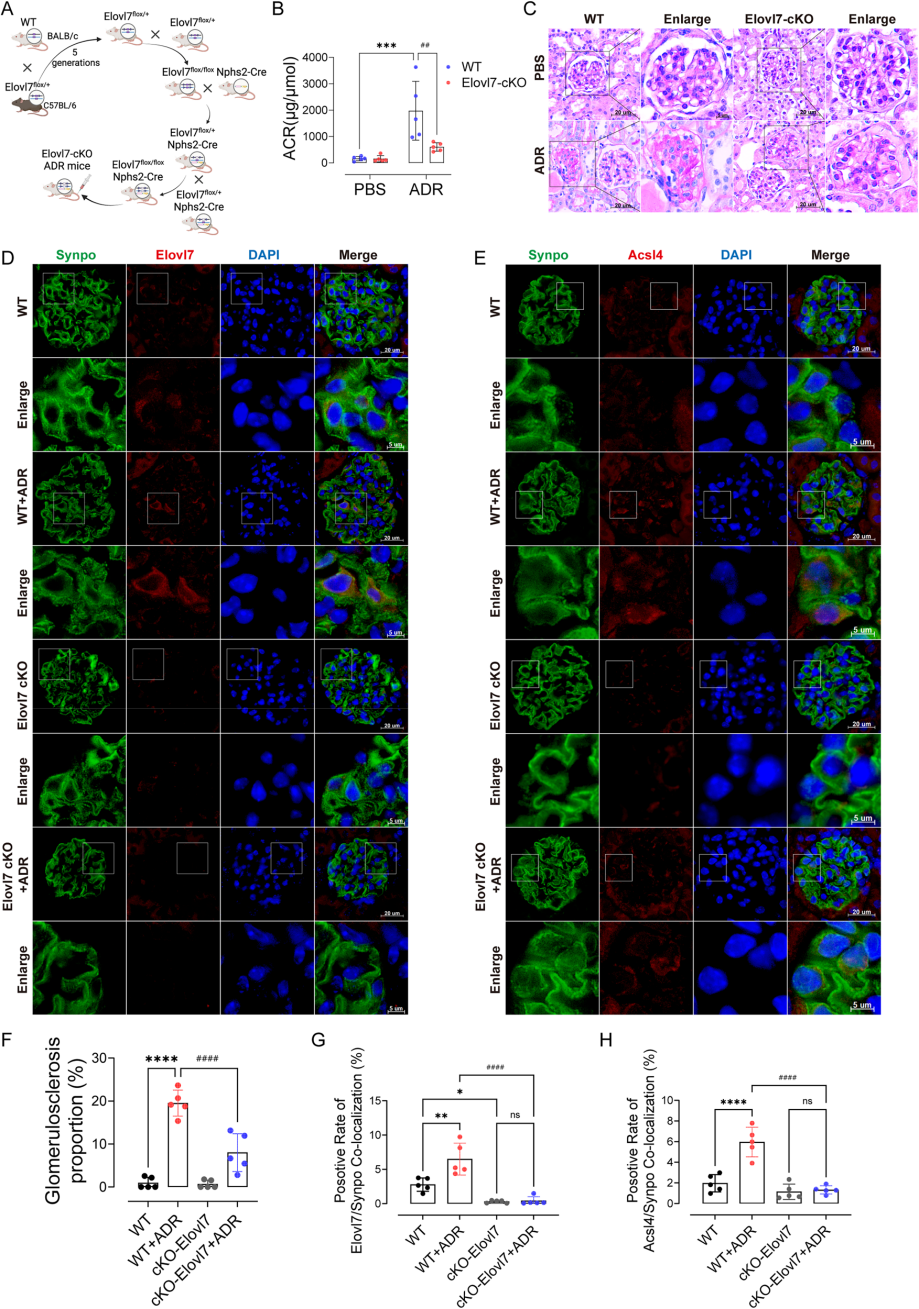
Conditional knockout of Elovl7 alleviated podocyte injury. **A** Breeding process of ELovl7^flox/flox^; Nphs2-Cre BAlB/c mice. **B** ACR of Elovl7-cKO mice 6 weeks after ADR administration (*n* = 5). Data are presented as mean ± SEM and statistical significance was assessed by one-way ANOVA followed by Tukey post-hoc test. ***p < 0.001 vs “WT + PBS” group; ^*##*^*p* < 0.01 vs “WT + ADR” group. **C** Representative PAS staining images of renal sections in WT or Elovl7-cKO mice with ADR administration (*n* = 5). **D** Immunofluorescence of Elovl7 co-localized with Synpo in WT or Elovl7-cKO mice with ADR administration (*n* = 5). **E** Immunofluorescence of Acsl4 co-localized with Synpo in WT or Elovl7-cKO mice with ADR administration (*n* = 5). **F** Glomerulosclerosis proportions in WT or Elovl7-cKO mice with ADR administration (*n* = 5). **G** Quantification of Elovl7 expression in podocytes by immunofluorescence co-localization in WT or Elovl7-cKO mice with ADR administration (*n* = 5). **H** Quantification of Acsl4 expression in podocytes by immunofluorescence co-localization in WT or Elovl7-cKO mice with ADR administration (*n* = 5). Data are presented as mean ± SEM and statistical significance was assessed by one-way ANOVA followed by Tukey post-hoc test. *p < 0.05, **p < 0.01, ****p < 0.0001, vs “WT” group; ^*###*^*p* < 0.001, ^*####*^*p* < 0.0001 vs “WT + ADR” group; ns not significant.

To further validate the role of Elovl7 in inducing podocyte injury through activating ferroptosis in vivo. We utilized mice with podocyte-specific knockout of Elovl7 by crossing Elovl7^flox/flox^ and Nphs2-Cre mice (Fig. 6A). Elovl7 knockout in podocytes reduced ADR-induced proteinuria (Fig. 6B), glomerular segmental sclerosis (Fig. 6C, F), and blocked the upregulated expression of Acsl4 in podocytes triggered by ADR administration (Fig. 6D, E, G, H).

### Fatty acid elongation abnormalities and ferroptosis are present in human podocytopathies

**Fig. 7:**
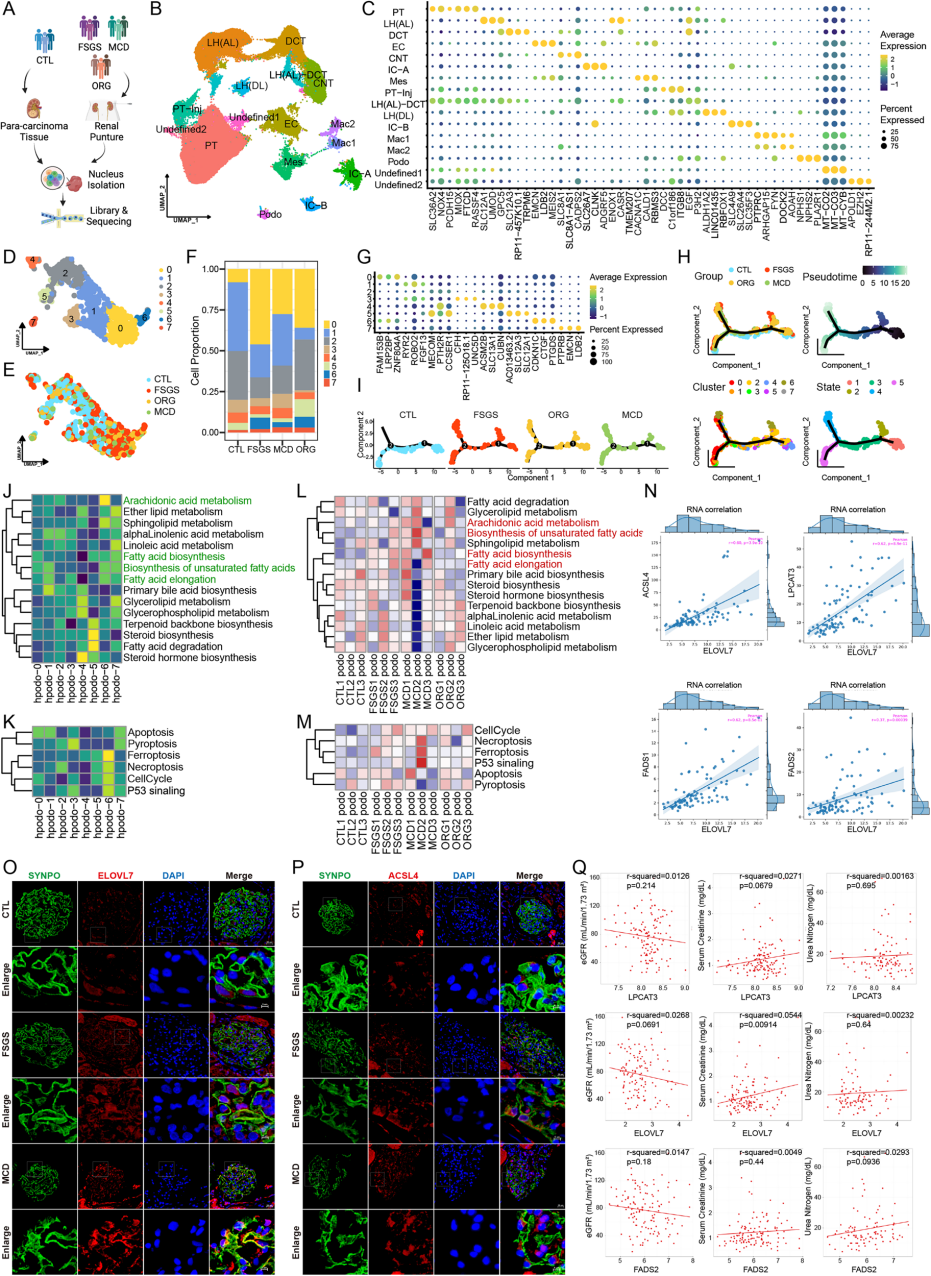
Fatty acid elongation disorder and ferroptosis exist in human podocytopathies. **A** Kidney biopsy tissues from FSGS, MCD and ORG patients and CTL kidney tissues were isolated for snRNA-seq (*n* = 3). **B** Umap plot visualizing 16 cell clusters from integrated kidney datasets. PT proximal tubule, PT-Inj injured proximal tubule, LH (AL) ascending limb of loop of Henle, DCT distal convoluted tubule, EC endothelial cell, CNT connecting tubule, IC-A type A intercalated cell of collecting duct, IC-B type B intercalated cell of collecting duct, Mes mesangial cell, LH (DL) descending limb of loop of Henle, Mac macrophage, Podo podocyte. **C** Dot plot showing specifically expressed genes in different types of cell clusters. **D** Umap plot visualizing 8 subclusters of podocytes. **E** Umap plot of integrated podocytes grouped by different diseased condition. **F** Stacked barplot show relative proportions of all subclusters of podocytes from FSGS, MCD and ORG patients. **G** Dot plot displaying specifically expressed genes in each subcluster of podocytes. **H** Pseudotime analysis of integrated podocytes with annotations of subcluster, state, disease group or peudotime. **I** Pseudotime plot of integrated podocytes split by different diseased group. **J** Heat map of gene module score of lipid metabolism pathways in different podocyte subclusters. **K** Heat map of gene module score of cell death pathways in different podocyte subclusters. **L** Heat map of gene module score of lipid metabolism pathways in podocytes from control or diseased groups. **M** Heat map of gene module score of cell death pathways in podocytes from control or diseased groups. **N** RNA correlations of *ELOVL7* and *ACSL4*, *LPCAT3*, *FADS1* and *FADS2* in kidney tissues from the GTEx. **O** Immunofluorescence of ELOVL7 co-localized with SYNPO in CTL, FSGS or MCD kidney sections from patients (*n* = 3). **P** Immunofluorescence of ACSL4 co-localized with SYNPO in CTL, FSGS or MCD kidney sections from patients (*n* = 3). **Q** Correlations between RNA levels of *LPCAT3*, *ELOVL7*, *FADS2* and eGFR, serum creatinine, urea nitrogen in glomerulopathies from the NEPTUNE.

To determine whether ferroptosis also plays a role in human podocytopathies, we analyzed snRNA-seq data for control and three types of glomerulopathy from adult kidney samples including FSGS, MCD and ORG (*n* = 3) (Fig. 7A). 16 cell types were annotated from 115,810 total cells by their highly expressed genes (Fig. 7B, C). Podocytes subclustering were then performed after removing the batch effect and 8 subclusters were defined (Fig. 7D–G). Specifically, the cell proportion of subset-6 arose in all three disease groups but not in the normal control group (Fig. 7F), suggesting a disease-associated cell state. Pseudotime analysis displayed cells of subset-6 appeared in State 2 and State 4, while cells in the normal control group exist in neither of the States (Fig. 7H, I), indicating the abnormal developmental trajectory of subset-6. Moreover, the gene module scores of *Fatty acid elongation*, *Biosynthesis of unsaturated fatty acids* and *Ferroptosis* were also higher in subset-6 than those in other subgroups (Fig. 7J, K). The activation of *Arachidonic acid metabolism* might be due to the accumulation of arachidonic acid. These LC-PUFA synthesis-related pathways and Ferroptosis were also activated to some extent in podocytopathy groups (Fig. 7L, M). Besides, *ELOVL7* showed positive correlation with several ferroptosis promoters (*ACSL4*, *LPCAT3*, *FADS1*, *FADS2*) in kidney tissues in the Genotype-Tissue Expression Project (GTEx) (Fig. 7N). Podocyte immunofluorescence co-localization showed increased expression of Elovl7 and Acsl4 in the podocytes of FSGS and MCD patients (*n* = 3) (Fig. 7O, P, Fig. S6g, h). In the Nephrotic Syndrome Study Network (NEPTUNE) database, LPCAT3, ELOVL7 and FADS2 were all negatively associated with eGFR and positively associated with creatine and urea nitrogen (Fig. 7Q). It is suggested that ferroptosis caused by disturbance of fatty acid elongation and unsaturated fatty acid synthesis pathway is also involved in human podocytopathies, which is consistent with our inference of podocyte injury in mice.

## Discussion

Podocyte injury and podocyte depletion are widely acknowledged as critical events in the initiation and advancement of glomerular diseases. Extensive podocyte injury can result in podocyte detachment and exposure of the basement membrane, leading to adhesion to Bowman’s capsule and eventually glomerulosclerosis [40, 41].

Recent advancements have significantly enhanced our understanding of kidney homeostasis through single-cell RNA sequencing (scRNA-seq). The high throughput of scRNA-seq has enabled a comprehensive understanding of cellular diversity and different subtypes or states of cells, revealing underlying cell characteristics in diseases [42]. However, limitations have hindered the widespread adoption of this transformative technology for podocyte analysis. Existing protocols often fail to accurately capture an adequate number of podocytes, as the dissociation harm these sensitive cells [43]. snRNA-seq has emerged as a promising alternative, showcasing comparable gene detection capabilities to scRNA-seq in the adult kidney. Moreover, snRNA-seq offers significant advantages, including reduced dissociation biases, compatibility with frozen samples, and the ability to yield a substantially higher number of podocytes compared to scRNA-seq [43].

In our snRNA-seq data of ADR kidney, we identified a population of injured podocytes with reduced expression of podocyte-specific genes, which is consistent with the view that podocyte injury is associated with reduction of podocyte markers such as NPHS1 and NPHS2 [44, 45]. For a long time, podocyte exhaustion has been primarily attributed to apoptosis [46, 47]. However, no apoptotic bodies were found in the electron microscope of glomerular diseases in vivo [48]. SnRNA-seq data from ADR-treated mice suggested that podocytes in the disease conditions exhibit a significantly higher ferroptosis score than those in control samples, indicating ferroptosis as a potential outcome of podocyte dysfunction. This hypothesis was further validated through experiments utilizing ferroptosis inhibitors both in vitro and in vivo. SnRNA-seq datasets of FSGS, MCD and ORG kidneys from patients also revealed a disease-associated subset of podocytes. This abnormal subcluster demonstrated heightened activities in *Fatty acid elongation*, *Biosynthesis of unsaturated fatty acid* and *Ferroptosis* compared to other podocyte subclusters.

Ferroptosis is a concomitant outcome of lipid metabolism disorder [14]. PUFAs are activated by Acsl4 and then incorporated into the phospholipid side chain by Lpcat3. The PUFA-PLs will become phospholipid radicals in the presence of oxygen radicals or peroxidase. These radicals can propagate to neighboring phospholipids via the Fenton reaction, ultimately producing phospholipid peroxides that contribute to ferroptosis. As the fatty acids at the sn1 position are typically SFAs or MUPAs, the level of saturation of the side chain at the sn-2 position determines the susceptibility of the PL to peroxidation. Targeted lipidomics analysis of podocytes revealed an increase in PLs with more than three double bonds (indicating at least one unsaturated fatty acyl tail) following ADR treatment. This suggests that podocytes in the ADR-treated group may be more prone to ferroptosis.

The diallyl group in double bond is sensitive to peroxidation. Theoretically, the susceptibility of the fatty acid to peroxidation is in proportion to the number of diallyl groups [32, 49], but the content of oxidized phospholipids in ADR-treated podocytes did not positively correlate with the degree of its tail unsaturation. This may require further validation.

Mammals are unable to synthesize fatty acids with double bonds at n-3 and n-6 positions due to lack of specific desaturases [50], such as linoleic acid (LA; 18:2n-6) and a-linolenic acid (ALA; 18:3n-3), hence these fatty acids must be acquired through diet. The 18-carbon precursor fatty acids undergo a series of desaturation and elongation reactions to produce longer, more unsaturated fatty acids [51, 52], referred as LC-PUFAs, such as arachidonic acid (AA; 20:4n-6) and docosahexaenoic acid (DHA; 22:6n-3), which are essential fatty acids for mammals [53, 54].

In addition to supporting normal physical development and biological function, the increase of PUFA in cells and their derived lipid peroxides increase the sensitivity to ferroptosis, as in conditions such as gastric cancer, acute liver failure and degenerative diseases [16, 55, 56]. On the contrary, monounsaturated fatty acids inhibit the occurrence and development of ferroptosis [57]. Fatty acids biosynthesis is dependent on fatty acid elongation enzymes and desaturases. Elovl5 promotes ferroptosis by increasing AA synthesis in a variety of tumor cells [58, 59]. FADS1 and FADS2 are also implicated in ferroptosis [60]. In our single-cell data, two elongases, Elovl6 and Elovl7, were found to be increased in podocytes. Elovl6, which is mainly involved in SFA and MUFA synthesis, was not found to be increased in our subsequent experiments, while Elovl7 was significantly increased both in vitro and in vivo models. It is reasonable to speculate that Elovl7 promotes the elevation of LC-PUFA and ferroptosis in podocytes. As Elovl7 is capable of elongating not only unsaturated fatty acids but also SFAs or MUFAs, we provided podocytes with three major substrates of Elovl7. As anticipated, γ-LA exacerbated ADR-induced podocyte injury and ferroptosis, indicating that Elovl7 enhances susceptibility to ferroptosis by elongating γ-LA to synthesize LC-PUFAs. This was further supported by experiments involving the knockdown of Elovl7 in vitro and its conditional knocked out in podocytes in vivo. Following ADR stimulation, LC-PUFA levels did not increase in Elovl7 knockdown MPC5 cells, and changes in cell viability and lipid peroxide markers were less pronounced compared to wide-type MPC5 cells. Moreover, the levels of albumin-to-creatinine ratio (ACR) and glomerular sclerosis induced by ADR were attenuated in Elovl7 conditional knockout mice.

While previous studies established ELOVL5’s role in promoting ferroptosis through arachidonic acid (AA) synthesis in tumor cells, our work demonstrates for the first time ELOVL7 (rather than ELOVL5) as the dominant elongase in podocyte ferroptosis. ELOVL7 uniquely elongates C18:3-derived LC-PUFAs. Our snRNA-seq approach enabled identification of disease-specific podocyte subpopulations (Fig. 7F), revealing that the ELOVL7 → LC-PUFA → ACSL4 axis constitutes a previously unrecognized pathway driving podocyte vulnerability to lipid peroxidation and glomerular disease progression.

Our study also reveals the role of podocyte ferroptosis in various podocytopathies. Single-nucleus RNA sequencing (snRNA-seq) revealed a disease-specific podocyte subset. Ferroptosis severity was greatest in FSGS, followed by MCD, and was least severe in ORG. ELOVL7 upregulation was most pronounced in FSGS compared to MCD and ORG. These observed differences align with known disease mechanisms: FSGS and MCD are characterized by extensive foot process effacement and severe podocyte injury, whereas ORG primarily shows glomerular hypertrophy with relatively preserved podocyte structure. These findings suggest that ferroptosis inhibition may be particularly relevant for FSGS treatment, while ORG might benefit more from metabolic interventions.

**Fig. 8:**
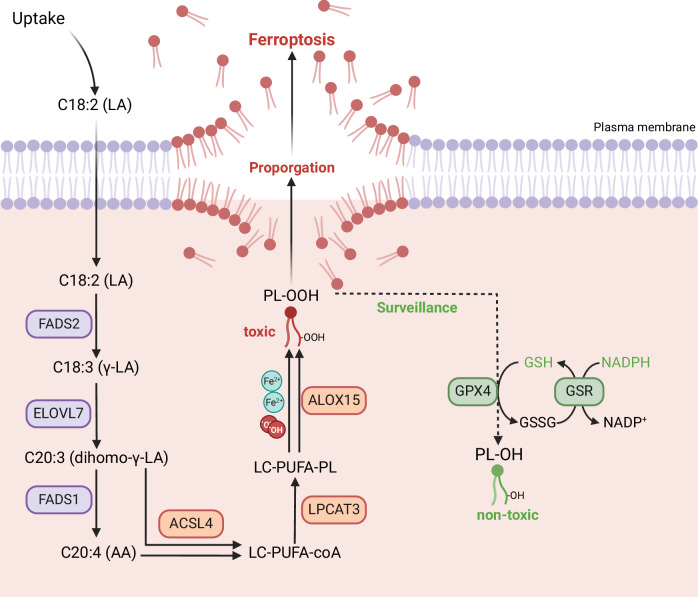
Mechanisms of podocyte injury in podocytopathy. In the disease state, the expression of several long-chain polyunsaturated fatty acid synthesis enzymes (*FADS2*, *ELOVL7*, *FADS1*) and ferroptosis-related genes (*ACSL4*, *LPCAT3*, *ALOX15*) is elevated, together with insufficient antioxidant substances (GSH, NADPH), lead to increased LC-PUFA synthesis from fed γ-LA through desaturation and elongation enzymes, and increased ferroptosis susceptibility. PL-OOH accumulates instead of being converted to nontoxic PL-OH, and the oxidation reaction is transmitted between adjacent phospholipids, eventually leading to membrane rupture and podocyte death.

In summary, we described that the abnormal expression of Elovl7 is involved in the disturbance of podocyte lipid metabolism, promotes the accumulation of LC-PUFAs and the remodeling of podocyte phospholipids, contributing to podocyte ferroptosis (Fig. 8). At present, there is a lack of direct inhibitors targeting ELOVL7 specificity. Our study provides the foundational evidence needed to drive ELOVL7-targeted drug development for podocytopathies.

## Conclusion

Our study visualized injured podocytes and substantial podocyte loss from multiple podocytopathies. This phenomenon could potentially be attributed to the increased synthesis of LC-PUFAs facilitated by Elovl7, which leads to accumulation of intracellular lipid peroxidation and ultimately leading to ferroptosis.

## Methods

### Animals and treatment

Wide-type (BALB/c) mice were obtained from the Animal Center of Sir Run Run Shaw Hospital, Zhejiang University School of Medicine. Nphs2-Cre (BALB/c) mice were a gift from Nanjing University. Elovl7^flox/+^ (C57BL/6) mice were purchased from Cyagen Bioscience (Suzhou, China). Elovl7^flox/+^ (BALB/c) were generated after Elovl7^flox/+^ (C57BL/6) crossed with Wide-type (BALB/c) mice more than five generations. Then Elovl7^flox/+^ (BALB/c) were mated with Elovl7^flox/+^ (BALB/c) to generate Elovl7^flox/flox^ (BALB/c) mice. Finally, Elovl7^flox/flox^ (BALB/c) were crossed with Nphs2-Cre (BALB/c) mice to obtain Podocyte specific Elovl7-deleted mice (Elovl7^flox/flox^; Nphs2-Cre (BALB/c) mice) (Fig. 6A). DNA extraction and genotyping were conducted using One Step Mouse Genotyping Kit (PD101-01, Vazyme). Littermates of wild-type or transgenic mice were used for the experiments. Genotypes were determined using the following primers (5’-3’):

flox-F1: TTGACTTGAATTTCTGGTTCACCC

flox-F2: CAAGTCATAAGTGGTAAAGCCCTG

flox-R2: TATGAGCAAGCACAACAAAGAACC

Cre-F: GGACATGTTCAGGGATCGCCAGGCG

Cre-R: GCATAACCAGTGAAACAGCATTGCTG

Podocyte injury mouse model was constructed using male BALB/c mice aged about eight weeks and weighting about 25 g. The species, strain, sex, age, body weight and health status of experimental mice were strictly controlled. The administration time of each experiment was controlled within one hour, the concentration and dose of the drug were strictly calculated according to the body weight of the mice, and the tail vein injection was completed by the same skilled experimenter. Different groups of mice in each experiment were housed in the same specific pathogen-free environment and adjacent cages with adequate food and water. Simple randomization (Random number remainder grouping method) was used to allocate each mouse to all groups. (1) For ADR mouse model establishment, twelve mice were randomly allocated to “CON” or “ADR” group (*n* = 6). Adriamycin (D1515, Sigma-Aldrich) was injected through the tail vein of mice (11 mg/kg) in ADR group; the same volume of PBS was injected into the tail vein of mice in CON group at the same speed. Most ADR mice develop proteinuria between 4 and 6 weeks, so the mice were weighed and urine was collected before treatment, after 3 weeks and 6 weeks respectively. Blood and bilateral kidneys were collected at the sixth week. One of the mice died three weeks after ADR administration, so it was excluded in measurements of ADR group at the sixth week. (2) For Fer-1 treatment experiment, twenty mice were randomly allocated to “CON + DMSO”, “ADR + DMSO”, “CON+Fer-1” or “ADR+Fer-1” group (*n* = 5). Fer-1 (HY-100579, MedChemExpress) was intraperitoneally injected (5 mg/kg) every other day after the administration of ADR. Mice in the corresponding control group were intraperitoneally injected with the same volume of DMSO. The mice were weighed and urine was collected at the sixth week. Blood and bilateral kidneys were also collected after 6 weeks. (3) For transgenic mice experiment, twenty mice were randomly allocated to “WT + PBS”, “cKO+PBS”, “WT + ADR”, “cKO+ADR” group (*n* = 5). ADR and PBS were administered in the same manner. Urine, blood, and bilateral kidneys were collected after 6 weeks. Both intraperitoneal and tail vein injections were sterilized with iodophor before injection, and compression hemostasis was performed after tail vein administration. The investigator was blinded to the group allocation during the experiments.

### Kidney samples from patients

Diseased kidney samples were renal biopsy tissues of patients diagnosed with FSGS, MCD or ORG from Department of Nephrology, Sir Run Run Shaw Hospital, Zhejiang University School of Medicine. The healthy control renal tissues were obtained from para-carcinoma tissues. Clinical information of patient samples is presented in Table S3. All patients signed an informed consent for sample collection.

### SnRNA-seq data analysis

#### Quality control of sequencing data and gene quantification

The library construction and sequencing data analysis were completed by Shanghai Ouyi Biomedical Technology Co., Ltd. The raw reads generated by high-throughput sequencing were in fastq format, and to ensure the sequencing quality, FastQC software was used to conduct quality control assessment on the raw data off the machine. The 10x genomics official software CellRanger was used to statistically analyze the data quality and align the reference genome for the raw data. This software identifies the Barcode sequence marker that distinguishes cells and the UMI marker for different mRNA molecules within each cell, allowing for quantitative analysis of high-throughput single-cell transcriptomes. The high-quality cell count, gene median, sequencing saturation, and other quality control statistical information were obtained.

#### Gene quantitative quality control and data preprocessing

Using the Seurat(v4.4.0) [61] package, we further quality control and process the data based on the initial quality control results obtained from Cellranger. In theory, most cells express a certain number of genes, have a certain number of UMIs, and express a certain level of mitochondrial genes, which are concentrated in a certain region. Based on this characteristic, we first filter out rogue cells by fitting a generalized linear model, and then filter and remove low-quality cells such as double cells, multicellular cells, or dead cells based on the distribution of nUMI, nGene, and percent.mito. Cells with gene number fewer than 200 or more than 5000, and cells with percentage of mitochondrial reads over 20% (Fig. S1a) were removed. Doublets were identified using DoubletFinder (v2.0.3) and 8% of the doublets were removed. Then data were logNormalized and scaled with the first 2000 high variable genes.

#### Dimension reduction and cluster analysis

Principal component analysis (PCA) was performed to linearly reduce the dimensionality of gene expression data, and the resulting PCA results were visualized in a two-dimensional space using t-distributed Stochastic Neighbor Embedding (tSNE) or Uniform Manifold Approximation and Projection (UMAP) (both nonlinear dimension reductions). Batch effects in single-cell expression profile data between samples were corrected by harmony (v1.2.0) [62].

#### Marker gene and cell type identification

Seurat [61] was utilized to identify differential up-regulation genes for each cell cluster relative to other cell populations. These identified genes represent potential markers for each cell cluster. The visualization of marker genes was achieved through DotPlot and VlnPlot functions.

#### Cell death scoring and metabolic pathway activity score

Single-cell module scores for cell death gene sets expression were performed with the AddModuleScore function in Seurat. Lipid metabolism gene sets scores were performed with ScMetabolism (v0.2.1) [63]. Then the mean module scores of podocytes in each sample were calculated and displayed with heat maps.

#### Pseudotime analysis

Podocytes pseudotime analysis was performed with monocle2 (v2.26.0) [64]. Briefly, we first extracted the expression matrix, phenoData and featureData of podocytes from the Seurat object to create a new CellDataSet object, then estimated the size factors and dispersions, and filtered low-quality cells, and finally selected the high variable genes for unsupervised analysis, sorted the cells, and completed the trajectory construction according to the trend of order gene expression.

#### Differential gene and enrichment analysis

Seurat [61] was used for differential gene screening, and significantly different genes were screened according to the conditions of *p* value less than 0.05 and difference multiple greater than 0.5. Ontology (GO) and Kyoto Encyclopedia of Genes and Genomes (KEGG) enrichment analysis of significantly different genes was performed by ClusterProfiler (v4.6.2) [65].

### Bulk RNA sequecing and analysis of differentially expressed genes

The libraries were sequenced on llumina Novaseq 6000 platform, generating 150 bp paired-end reads. The fastp [66] was employed to process the raw reads in fastq format, resulting in clean reads after eliminating low-quality sequences for subsequent data analysis. Reference genome alignment was performed using HISAT2 [67]. FPKM [68] and read counts of each gene were obtained using HTSeq-count [69]. PCA analysis and were carried out in R (v3.2.0) to assess biological replicates of the samples. DESeq2 [70] was utilized for differential expression analysis, defining genes with a q value < 0.05 and foldchange > 2 or foldchange < 0.5 as DEGs.

### Deconvolution analysis

Using music2_prop_t_statistics function in MuSiC2 (v0.1.0) [71], the kidney bulk transcriptome matrix splitted by phenotype was decomposed to obtain the fraction of single-cell subsets in each group.

### Determination of proteinuria in mice

Mouse albuminuria was detected using Mouse Microalbuminuria ELISA kit (E-EL-M0792, Elabscience), along with urinary creatinine (C011-2-1, Jiancheng Bio, Nanjing, China) for standardization.

### Pathological and immunofluorescence staining

Kidney tissue was harvested and fixed overnight in 4% paraformaldehyde, thendehydrated in an automatic dehydrator and embedded in paraffin. The tissue wax blocks were cut into 3.5 μm slices, then dewaxed and hydrated.

For Periodic Acid-Schiff Staining (PAS) staining, the sections were oxidized in periodate solution, then placed in Schiff reagent, differntiated with stannous sulfate solution, counterstained with haematoxylin, and finally dehydrated, transparent and sealed with neutral tree glue.

For immunofluorescent co-localization staining, the antigens were repaired with sodium citrate-EDTA repair solution by microwave for 15 min. Sections were blocked with 5% BSA for 1 h at room temperature. Both primary antibodies (Synpo (sc-515842, Santa Cruz Biotechnology, Texas, USA) with Acsl4 (A20414, ABclonal, Wuhan, China) or Elovl7 (PA5-20913, Invitrogen, California, USA)) were incubated overnight at 4 °C simultaneously, followed by both Alexa Fluor™ 488 Goat Anti-Mouse IgG antibody (A-11001, Invitrogen) and Alexa Fluor™ 568 Goat Anti-Rabbit IgG antibody (A-11011, Invitrogen). Finally, the plates were sealed with anti-quenching sealing tablet (P36935, Invitrogen) and photographed by fluorescence microscopy. ImageJ (v1.54) was used for immunofluorescence co-localization and quantitative analysis. The investigator was blinded to the group allocation during the experiment.

### Cell culture

MPC5 cells was cultured in 1640 medium supplemented with 10% fetal bovine serum, 100 μg/ml streptomycin and 10 U/mL recombinant mouse γ-interferon (γ-IFN, 391275, Cell Signaling Technology, Boston, USA) at 33 °C for proliferation and at 37 °C in the absence of γ-IFN for differentiation for two weeks. Well differentiated cells were then used for next experiments. All experiments in this study were performed using cells that had undergone STR profiling and were confirmed to be mycoplasma-free.

### Establishment of podocyte damage model in vitro

MPC5 cells were stimulated with 1%FBS medium supplemented with Adriamycin (8 μg/ml) for 24 h, and follow-up tests were performed.

### SiRNA transfection

Cells were seeded in 6-well plates and siRNA was transfected the next day for 48 h using Lipofectamine 3000 (L3000008, Thermo Scientific, Massachusetts, USA) according to the manufacturer’s instructions.

### Cell survival assay

The cells were implanted in 96-well plates with a density of 1000/ well. After treatment, the treated medium was changed to serum-free medium and 10ul cck8 (K1018, Apex Bio, Houston, USA) was added to each well and incubated at 37 °C for 1–2 h. Then the absorbance at 450 nm was measured with an enzyme labeler.

### Cellular MDA, GSH, NADPH assay

Cells were seeded in 6-well plates, stimulation was performed when the degree of cell fusion reached 60–70%. Cells were lysed and the levels of MDA, GSH, and NADPH were detected according to the instructions of MDA Assay Kit (M496, Dojindo, Kumamoto, Japan), GSH and GSSG detection kit (S0053, Beyotime, Shanghai, China), NADP^+^/NADPH detection kit (S0179, Beyotime) respectively. Cellular protein concentration was also measured by BCA Protein concentration Assay kit (P0012, Beyotime) for MDA and NADPH correction.

### Mitochondrial ROS and lipid peroxide detection in cells

MPC5 cells were planted in confocal dishes and treated when the cell fusion reached 60–70%, cells were stained with MitoSOX (RM02822, ABclonal) to detect mitochondrial ROS or Liperfluo (L248, Dojindo) to detect lipid peroxides after stimulation. The images were observed and captured using confocal microscopy.

### Flow cytometry

For the detection of Liperfluo, the cells were seeded in a medium dish. After the cells in each group were treated, the medium was removed, cells were washed with PBS and added with 1μmol/L Liperfluo solution. The cells were cultured at 37 °C in a 5% CO_2_ incubator for 30 min, and the solution was removed and washed twice with PBS. After trypsin digestion, the cells were resuspended in PBS and analyzed by flow cytometry using CytoFLEX S (Beckman Coulter, California, USA).

For apoptosis detection, 10^6^ treated cells were collected from each group, and the level of apoptosis in each group and detected by CytoFLEX S flow cytometry using Annexin V-APC/7-AAD apoptosis kit (Multi Sciences, Shanghai, China).

### Quantitative reverse transcription-polymerase chain reaction (qRT-PCR)

Total intracellular RNA was extracted using FastPure Cell/Tissue Total RNA Isolation Kit (RC112-01, Vazyme, Nanjing, China). HiScriptⅢ 1st Strand cDNA Synthesis Kit (R312-02, Vazyme) was used to reverse transcribe RNA into cDNA. Quantitative PCR was carried out in the QuantStudio™ 6 Flex Real-Time PCR System (Applied Biosystems, Massachusetts, USA) using SYBR Green (Q711-03, Vazyme). Primer sequences are listed in Table S2. RNA expressions were normalized to β-actin and were analyzed through ΔΔCt method.

### Western blotting

Protein was extracted from cell samples on ice using lysis buffer (P0013, Beyotime) containing PMSF. All proteins were denatured by a metal bath at 95 °C for 5 min except Elovl7 (sample heating affect the detection of elongase [72]). The protein lysates were separated by SDS-PAGE, followed by transferring onto 0.45 μm PVDF membrane (Merck Millipore, Darmstadt, Germany). Primary antibodies against Acsl4 (A20414, ABclonal), Lpcat3 (ABclonal, A17604), Elovl7 (PA5-20913, Invitrogen), β-actin (ZB15001-hrp-100, Servicebio, Wuhan, China) were incubated overnight at 4 °C and secondary antibodies conjugating horseradish peroxidase (HRP) (SA00001-2, Proteintech, Wuhan, China) were applied for 1 h at room temperature. Protein blots were visualized under Fusion FX Chemiluminescence Imaging System (Vilber GmbH, Paris, France). Semi-quantifications of protein bands were analyzed using ImageJ (v1.54). The full length uncropped original western blots were showed in Fig. S7.

### Lipid extraction

Lipids were extracted from approximately one million cells using a modified version of the Bligh and Dyer’s method as described previously [73]. Briefly, cells were homogenized in 750 µL of chloroform: methanol: MilliQ H_2_O (3:6:1) (v/v/v). The homogenate was then incubated at 1500 rpm for 1 h at 4 °C. At the end of the incubation, 350 µL of deionized water and 250 µL of chloroform were added to induce phase separation. The samples were then centrifuged and the lower organic phase containing lipids was extracted into a clean tube. Lipid extraction was repeated once by adding 450 µL of chloroform to the remaining cells in aqueous phase, and the lipid extracts were pooled into a single tube and dried in the SpeedVac under OH mode. Samples were stored at −80 °C until further analysis. Upper aqueous phase and cell pellet were dried in a SpeedVac under H_2_O mode. Total protein content was determined from the dried pellet using the Pierce® BCA Protein Assay Kit according to the manufacturer’s protocol.

### Lipidomics analyses

Lipidomic analyses were conducted at LipidALL Technologies using a Shimadzu Nexera 20AD-HPLC coupled with Sciex QTRAP 6500 PLUS as reported previously [74]. Separation of individual lipid classes of polar lipids by normal phase (NP)-HPLC was carried out using a TUP-HB silica column (i.d. 150 × 2.1 mm, 3 µm) with the following conditions: mobile phase A (chloroform: methanol: ammonium hydroxide, 89.5:10:0.5) and mobile phase B (chloroform: methanol: ammonium hydroxide: water, 55:39:0.5:5.5). MRM transitions were set up for comparative analysis of various polar lipids. Individual lipid species were quantified by referencing to spiked internal standards. d9-PC32:0(16:0/16:0), d9-PC36:1p(18:0p/18:1), d7-PE33:1(15:0/18:1), d9-PE36:1p(18:0p/18:1), d31-PS(d31-16:0/18:1), d7-PA33:1(15:0/18:1), d7-PG33:1(15:0/18:1), d7-PI33:1(15:0/18:1), d7-LPE18:1, C17-LPI, C17-LPA, C17-LPS, were obtained from Avanti Polar Lipids. GM3-d18:1/18:0-d3 was purchased from Matreya LLC. Free fatty acids were quantitated using d31-16:0 (Sigma-Aldrich) and d8-20:4 (Cayman Chemicals).

### Fatty acid flux analysis

Fatty acid flux analysis was conducted at LipidALL Technologies as described previously [75]. Lipids were extracted from cells using 1 mL of ice-cold methanol. Samples were incubated at 1500 rpm for 30 min at 4 °C. At the end of the incubation, samples were centrifuged for 10 min at 12,000 rpm at 4 °C. Clean supernatant was transferred to a new tube and dried in a SpeedVac under OH mode. Total protein content was determined from the dried pellet using the Pierce® BCA Protein Assay Kit according to the manufacturer’s protocol. The dried extract was reconstituted in LCMS grade methanol for fatty acid flux analysis on a system comprising an Agilent 1290 II UPLC coupled to Sciex 5600+ quadrupole-TOF MS. Fatty acids were separated on a Waters ACQUITY HSS-T3 column (3.0 × 100 mm, 1.8 μm). MS parameters for detection were: ESI source voltage negative ion mode −4.5 kV; vaporizer temperature, 500 °C; drying gas (N2) pressure, 50 psi; nebulizer gas (N2) pressure, 50 psi; curtain gas (N2) pressure, 35 psi [76]. Information-dependent acquisition mode was used for MS/MS analyses of the metabolites. Collision energy was set at −30 ± 15 eV. Data acquisition and processing were performed using Analyst® TF 1.7.1 Software (AB Sciex, Concord, ON, Canada). All detected ions were extracted using MarkerView 1.3 (AB Sciex, Concord, ON, Canada) into Excel in the format of two dimensional matrix, including mass to charge ratio (m/z), retention time and peak areas. PeakView 2.2 (AB Sciex, Concord, ON, Canada) was applied to extract MS/MS data and perform comparisons with the Metabolites database (AB Sciex, Concord, ON, Canada), HMDB and standard references to annotate ion identities [77]. 19:0-FFA was used as the internal standard to correct the endogenous metabolites in the samples and normalize them with protein amounts.

### Preparation of fatty acid-enriched cells

The cells were placed in a medium containing 1%FBS with BSA-conjugated γ-Linolenic acid (γ-LA, HY-N7140, MedChemExpress, New Jersey, USA), Oleic acid (OA, HY-N1446, MedChemExpress) or Stearic acid (SA, HY-B2219, MedChemExpress) for 24 hours (80 μM) with or without adriamycin (8 μg/ml).

### Cellular oil red staining

The treated cells were washed three times with PBS and fixed with 4% paraformaldehyde for 10 minutes. Then covering 60% isopropanol within cells for 20 s. Oil red O staining solution (BL941A, Biosharp, Hefei, China) was added and removed after 30 min of dark staining at room temperature. Cells were covered with 60% isopropanol for rapid differentiation and washed 3 times with pure water. Finally, nuclei were stained with hematoxylin for 1 min. After washing with water and adding PBS in well plate, cells were observed with an inverted microscope.

### Statistical analysis

Statistical analysis for cell line and animal studies was carried out by GraphPad Prism (v8.3.0). Test for normal distribution was performed using Shapiro-Wilk test. All data are presented as mean ± standard error of the mean (SEM). Significance was measured by two-tailed unpaired Student’s *t* test for comparison between two groups and one-way ANOVA followed by Tukey post-hoc test for multiple group comparison.

## Supplementary information

Figure S1

Figure S2

Figure S3

Figure S4

Figure S5

Figure S6

Figure S7

Supplementary Legends

Table S1

Table S2

Table S3

## Data Availability

The snRNA-seq data supporting the findings of this study and the code used in the analyses are openly available in Zenodo (10.5072/zenodo.106900). SnRNA-seq data of KDKD podocytes data was retrieved from GEO (GSE164274). The RNA correlation data were derived from the Genotype-Tissue Expression Program (GTEx): https://commonfund.nih.gov/GTEx. The Correlation between RNA levels and eGFR, serum creatinine, urea nitrogen in glomerulopathies were derived from the Nephrotic Syndrome Study Network (NEPTUNE): https://www.neptune-study.org/. Targeted lipidomics data are presented in Table S3.
