# Supplementary figures and images for: Elovl7 sensitizes podocytes to ferroptosis in podocytopathy by elongating polyunsaturated fatty acids
Source: Cell Death Dis. 2025 Nov 24;16(1):857. doi: 10.1038/s41419-025-08144-4 (PMC12644464; doi:10.1038/s41419-025-08144-4)

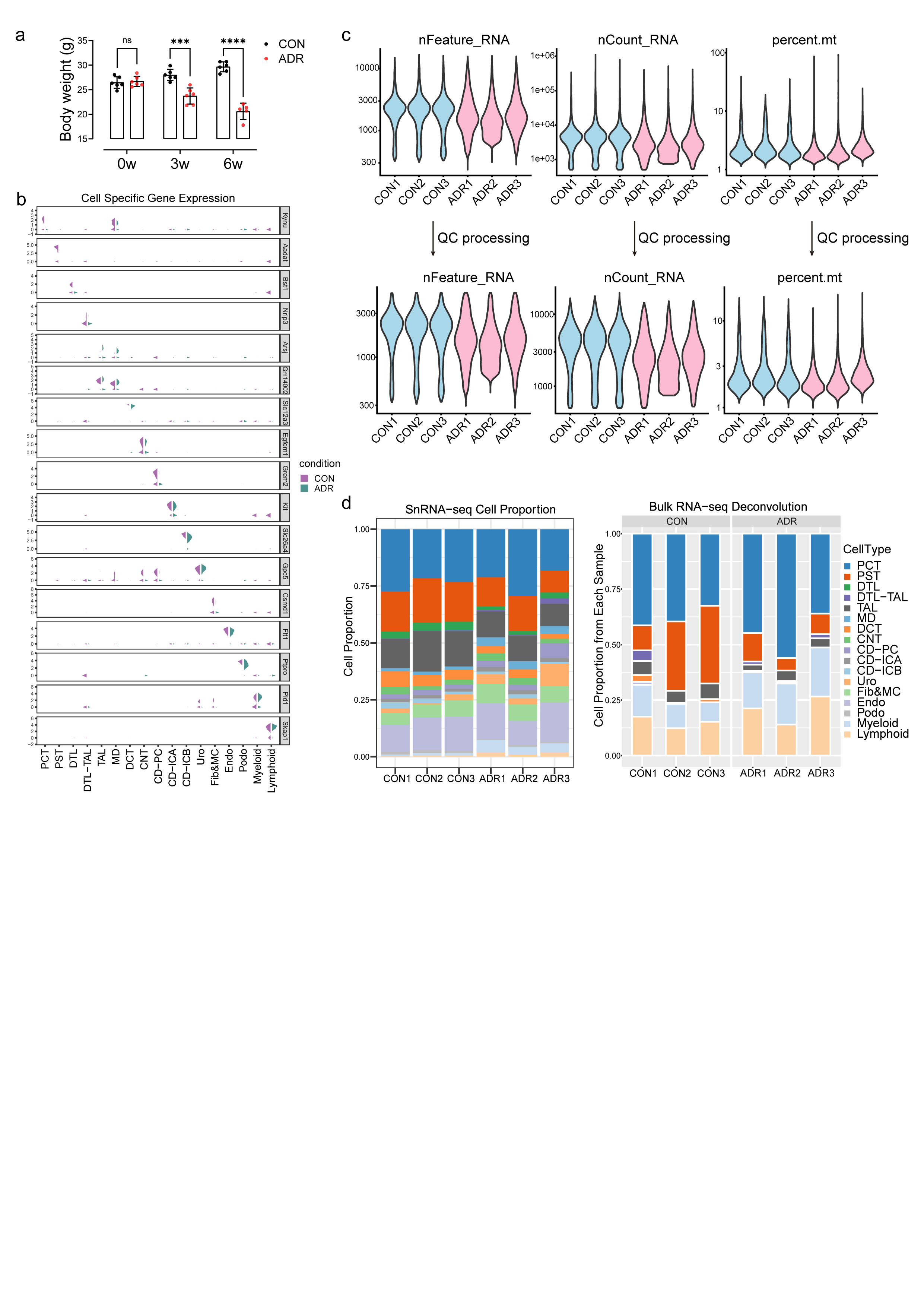

Supplement: Supplementary file 1 — Figure S1 [file 41419_2025_8144_MOESM1_ESM.png]

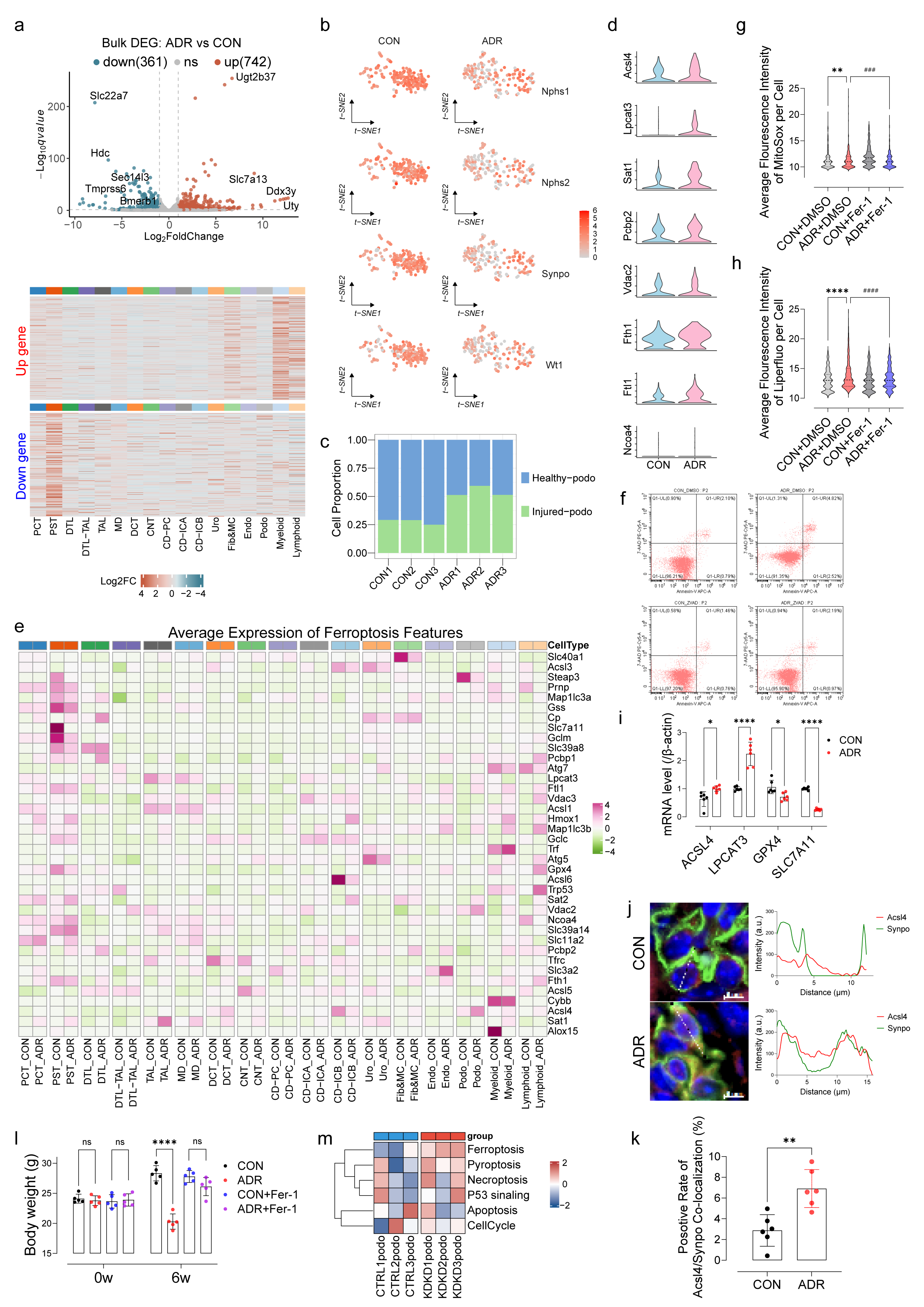

Supplement: Supplementary file 2 — Figure S2 [file 41419_2025_8144_MOESM2_ESM.png]

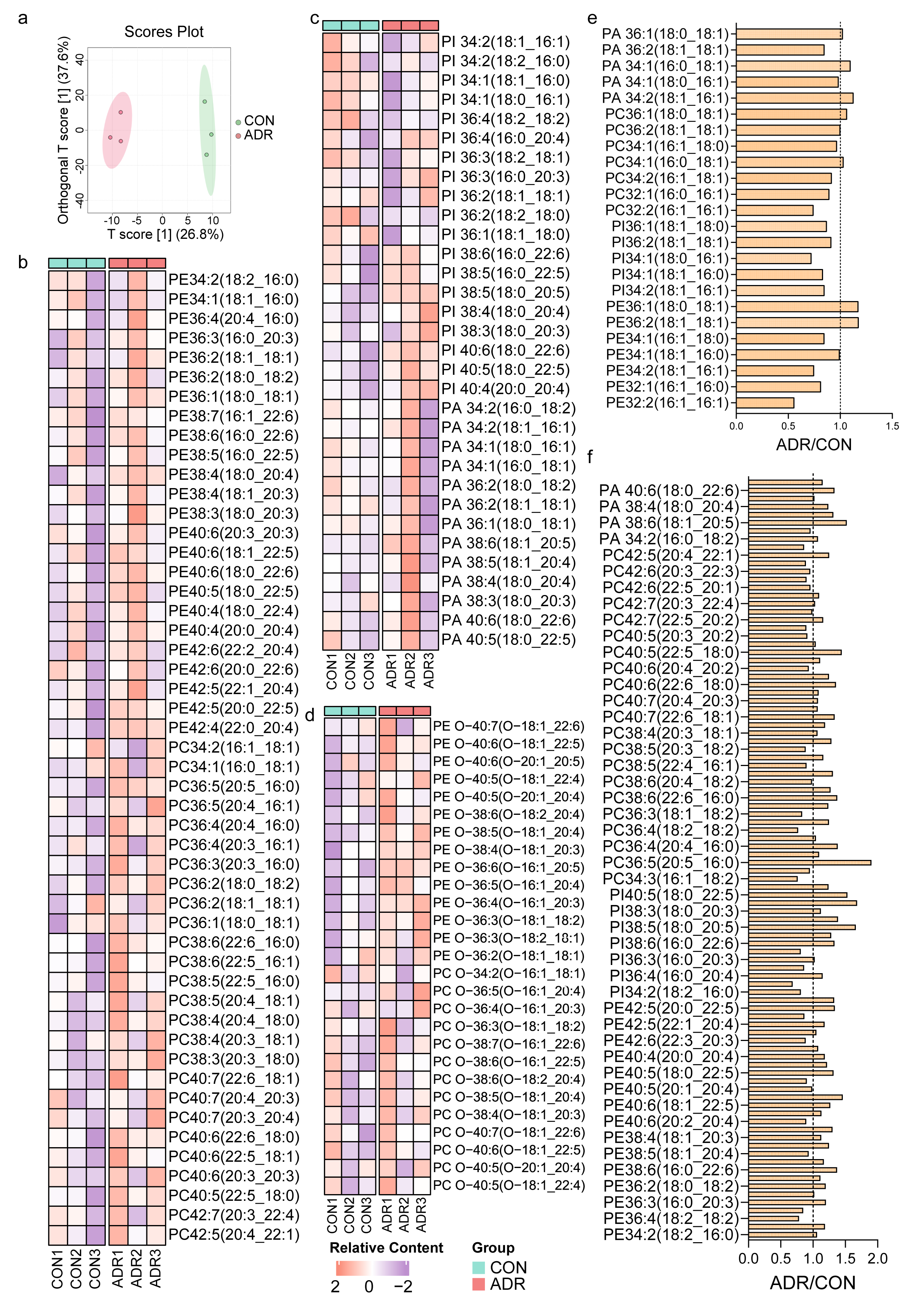

Supplement: Supplementary file 3 — Figure S3 [file 41419_2025_8144_MOESM3_ESM.png]

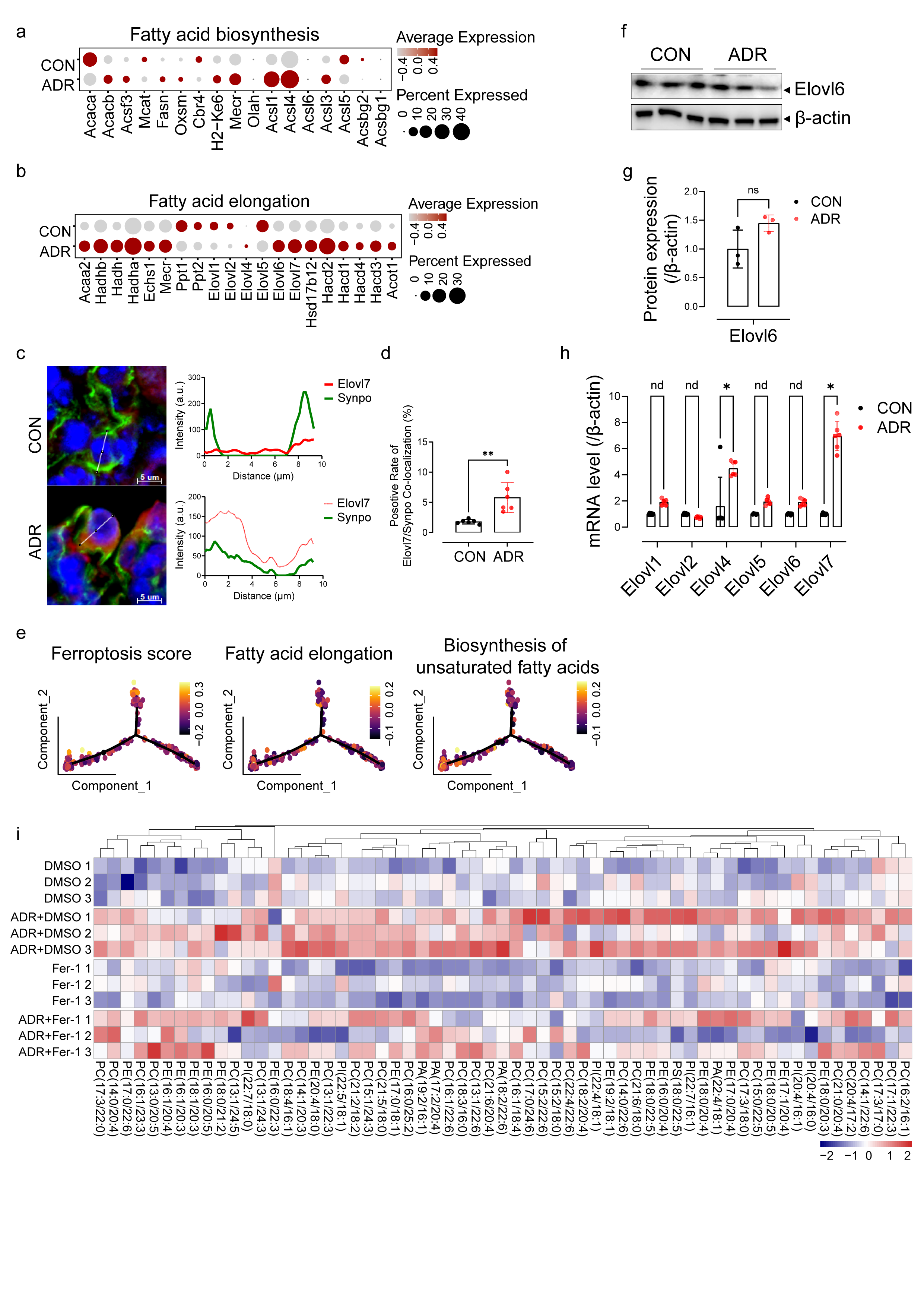

Supplement: Supplementary file 4 — Figure S4 [file 41419_2025_8144_MOESM4_ESM.png]

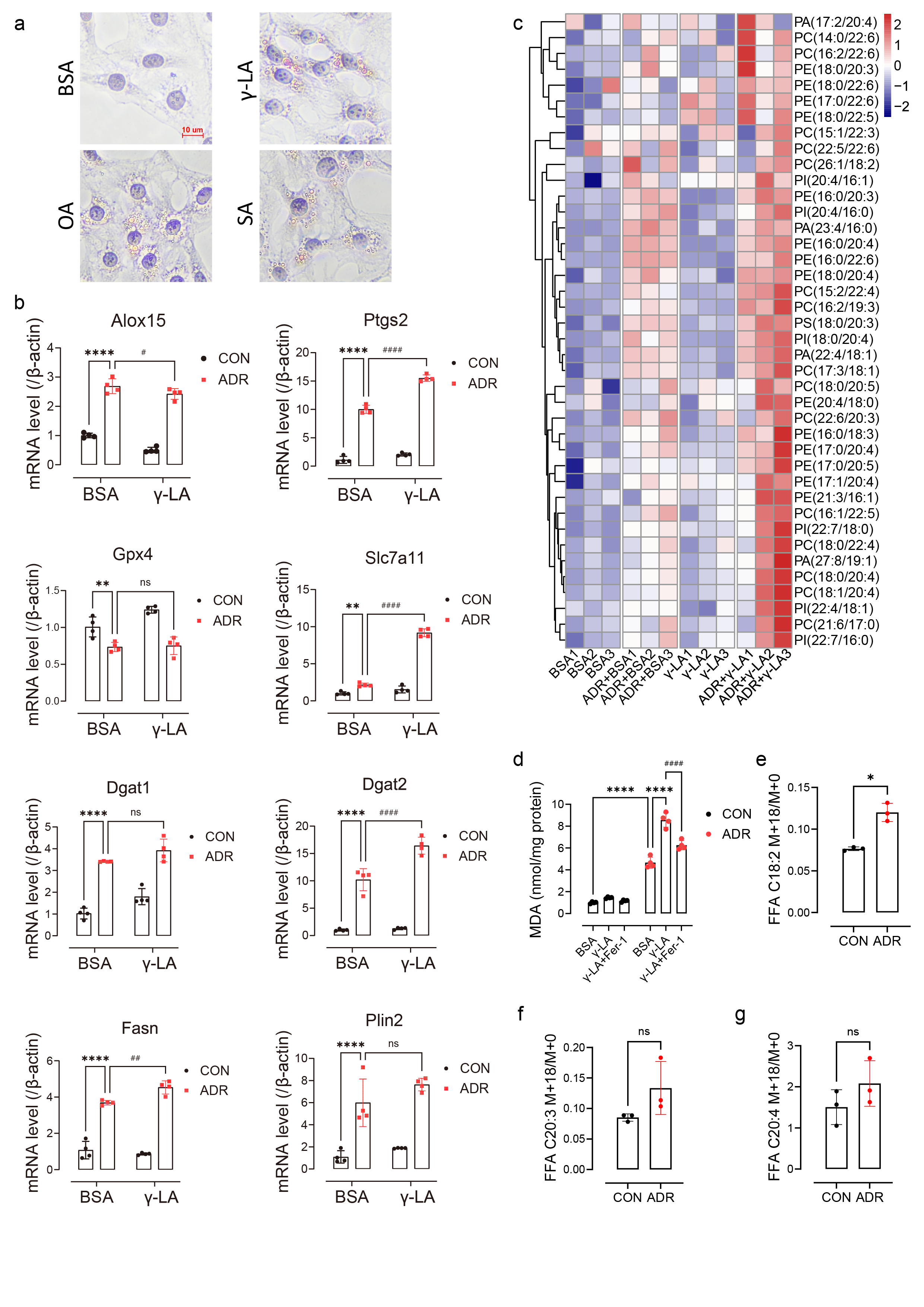

Supplement: Supplementary file 5 — Figure S5 [file 41419_2025_8144_MOESM5_ESM.png]

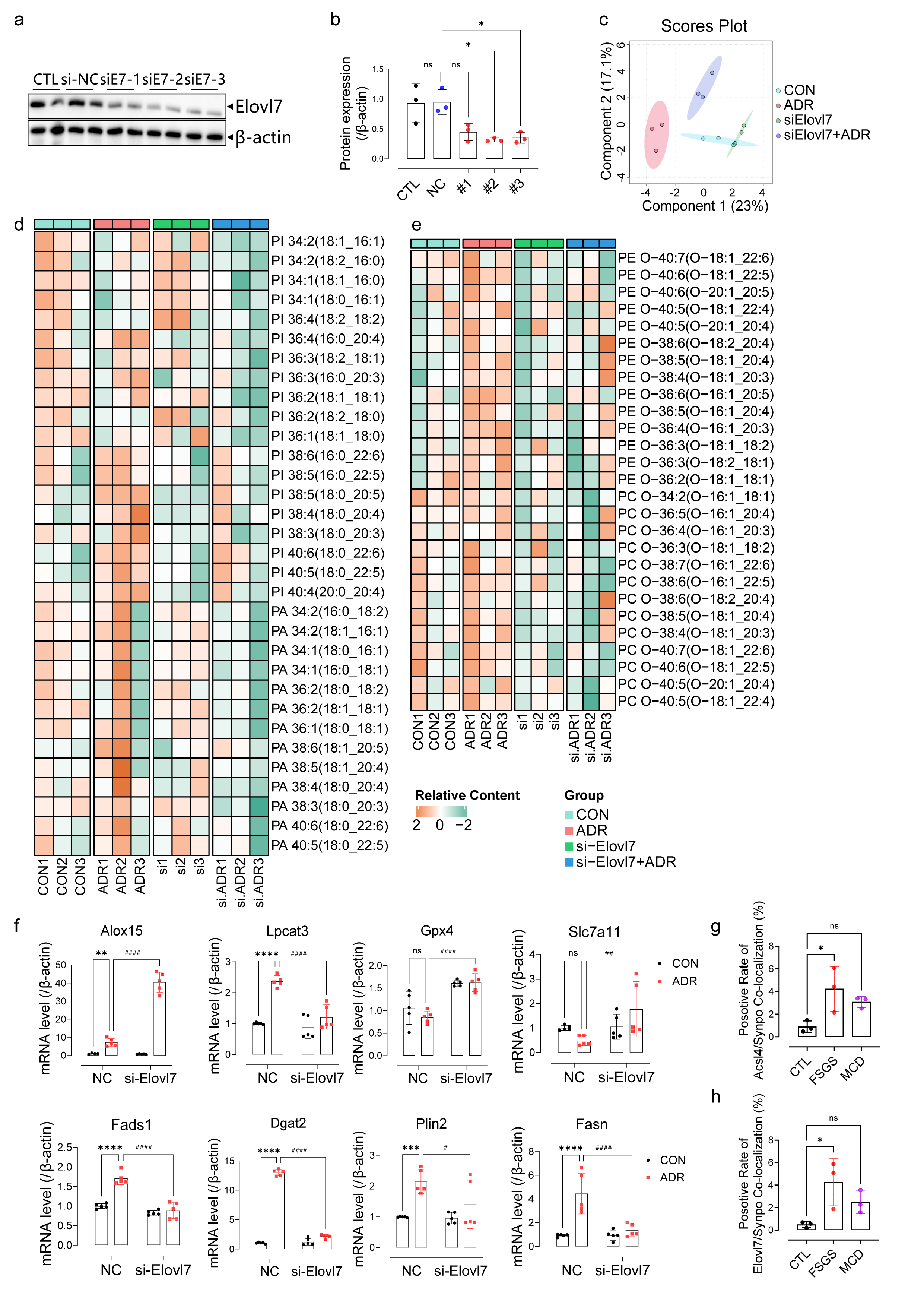

Supplement: Supplementary file 6 — Figure S6 [file 41419_2025_8144_MOESM6_ESM.png]

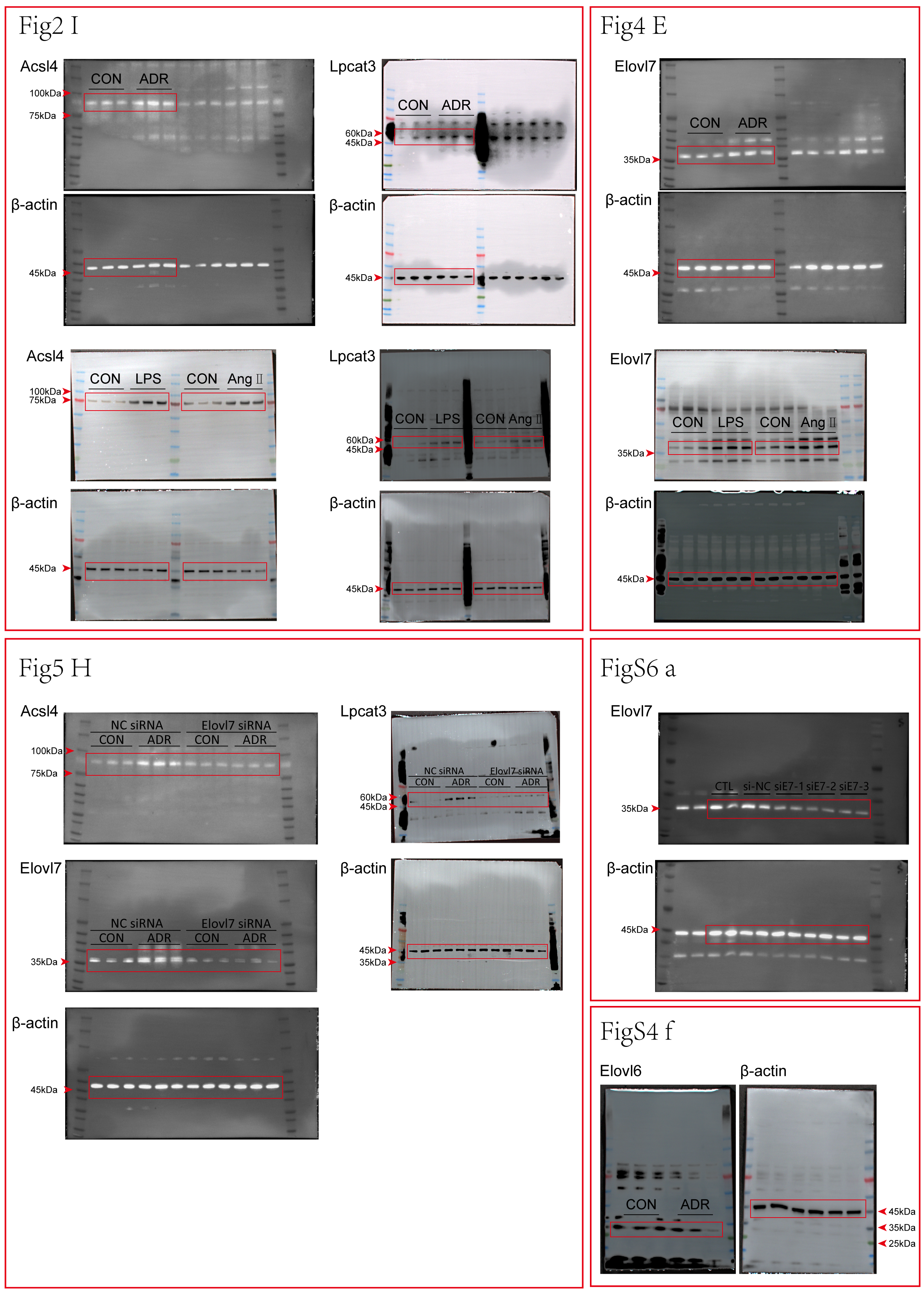

Supplement: Supplementary file 7 — Figure S7 [file 41419_2025_8144_MOESM7_ESM.png]
