# Supplementary material for: Elovl7 sensitizes podocytes to ferroptosis in podocytopathy by elongating polyunsaturated fatty acids
Source: Cell Death Dis. 2025 Nov 24;16(1):857. doi: 10.1038/s41419-025-08144-4 (PMC12644464; doi:10.1038/s41419-025-08144-4)
Supplement: Supplementary file 8 — Supplementary Legends [file 41419_2025_8144_MOESM8_ESM.docx]

**Fig.S1. Podocytes were significantly damaged in ADR group**

1. Body weights of ADR mice at 0w, 3w and 6w (*n* = 6). Data are presented as mean ± SEM and statistical significance was assessed by two-tailed unpaired Student’s t-test. ****p* < 0.001, *****p* < 0.0001 vs CON group; ns, not significant.
2. Violin plot showing expression of cell type-specific genes between CON and ADR group in snRNA-seq data.
3. The numbers of feature, count and percentage of mitochondrial reads in each cell of snRNA-seq data before and after filtering.
4. Staked bar plot showing proportion of each cell cluster in snRNA-seq dataset and bulk RNA-seq deconvolution of ADR mice between different samples.

**Fig.S2. Specific ferroptosis occurred in injured podocytes**

1. DEGs between ADR and CON group in bulk RNA-seq and their expression in different cell type in snRNA-seq data.
2. Feature plot showing expression of specific podocyte markers in podocytes split by group.
3. Stacked bar chart showing cell proportion of podocyte subclusters in different samples.
4. Violin plot showing expression of genes involved in ferroptosis in podocytes split by group.
5. Heatmap showing expression level of genes promoting ferroptosis in each cell type in CON or ADR group in snRNA-seq data.
6. Cell apoptosis was detected by flow cytometry.
7. Quantification of MitoSox in podocytes treated in different condition.
8. Quantification of MitoSox in podocytes treated in different condition.

Data are presented as mean ± SEM and statistical significance was assessed by one-way ANOVA followed by Tukey post-hoc test. ***p* < 0.01, *****p* < 0.0001 vs “CON+DMSO” group; *^###^p* < 0.001, *^####^p* < 0.0001 vs “ADR+DMSO” group.

1. mRNA expression of genes related to ferroptosis in ADR treated MPC5 cells (*n* = 6). Data are presented as mean ± SEM and statistical significance was assessed by two-tailed unpaired Student’s *t*-test. **p* < 0.05, *****p* < 0.0001 vs CON group.
2. Immunofluorescence co-localization analysis of Fig2L.
3. Quantification of Acsl4 expression in podocytes by immunofluorescence co-localization (*n* = 6). Data are presented as mean ± SEM and statistical significance was assessed by two-tailed unpaired Student’s *t*-test. ***p* < 0.01 vs CON group.
4. Body weights of ADR mice with or without Fer-1 treatment at 0w or 6w (*n* = 5). Data are presented as mean ± SEM and statistical significance was assessed by one-way ANOVA followed by Tukey post-hoc test. *****p* < 0.0001 vs CON group; ns, not significant for any comparison.
5. Heatmap showing means of gene module score of cell death pathways for podocytes in CTRL or KDKD group of snRNA-seq data from GEO (GSE164274).

**Fig.S3. Elovl7 mediated the accumulation of PLs with LC-PUFAs in ADR group**

1. OPLS-DA analysis of targeted lipidomics for CON or ADR-treated MPC5 cells. Each point represents a sample (*n* = 3). Ellipses represent 95% confidence regions.
2. Heatmap showing relative contents of PE and PC with tails of different length and saturation in CON or ADR-treated cells.
3. Heatmap showing relative contents of PI and PA with tails of different length and saturation in CON or ADR-treated cells.
4. Heatmap showing relative contents of oxidative PLs in CON or ADR-treated cells.
5. SFA and MUFA-PLs content ratio between ADR and CON group.
6. PUFA-PLs content ratio between ADR and CON group.

**Fig.S4. Elovl7 expression levels were specifically increased in the ADR group**

1. Dot plot showing expression of genes involved in the *Fatty acid biosynthesis* pathway in podocytes of snRNA-seq data.
2. Dot plot showing expression of genes involved in the *Fatty acid elongation* pathway in podocytes of snRNA-seq data.
3. Immunofluorescence co-localization analysis of Fig4G.
4. Quantification of Elovl7 expression in podocytes by immunofluorescence co-localization (*n* = 6). Data are presented as mean ± SEM and statistical significance was assessed by two-tailed unpaired Student’s *t*-test. ***p* < 0.01 vs CON group.
5. Module scores of *Ferroptosis*, *Fatty acid elongation* and *Biosynthesis of unsaturated fatty acids* along the pseudotime trajectory of podocytes.
6. Immunoblots of Elovl6 in MPC5 cells treated by ADR (*n* = 3).
7. Quantification of Elovl6 blots normalized to β-actin for f (*n* = 3). Data are presented as mean ± SEM and statistical significance was assessed by two-tailed unpaired Student’s t-test. ns, not significant.
8. mRNA expression of elongase genes in ADR treated MPC5 cells (*n* = 6). Data are presented as mean ± SEM and statistical significance was assessed by two-tailed unpaired Student’s t-test. *p < 0.05 vs CON group; nd, not significant.
9. Heatmap showing relative contents of PUFA-PLs in ADR stimulated cells with or without Fer-1 treatment.

**Fig.S5. γ-LA supplementation enhanced ADR-induced lipid metabolism disorder in podocyte**

1. Representative images of oil red staining in γ-LA, OA or SA stimulated MPC5 cells (*n* = 3).
2. mRNA expression of genes related to ferroptosis (*Alox15*, *Ptgs2*, *Gpx4*, *Slc7a11*), triacylglycerol synthesis (*Plin2*, Dgat1, *Dgat2*) and de novo synthesis (*Fasn*) (*n* = 4). Data are presented as mean ± SEM and statistical significance was assessed by one-way ANOVA followed by Tukey post-hoc test. ***p* < 0.01, *****p* < 0.0001 vs “CON+BSA” group; *^##^p* < 0.01, *^####^p* < 0.0001 vs “ADR + BSA” group; ns, not significant.
3. Heatmap showing relative contents of PUFA-PLs in ADR stimulated cells with or without γ-LA treatment.
4. Content of MDA in CON or ADR cells with γ-LA and Fer-1 treatment (*n* = 4). Data are presented as mean ± SEM and statistical significance was assessed by one-way ANOVA followed by Tukey post-hoc test.  *****p* < 0.0001 vs “ADR + BSA” group; *^####^p* < 0.0001 vs “ADR + γ-LA” group.

e-f. Relative enrichment of the labeled (m + 18) fatty acids (FFAs) in cells cultured in medium containing ¹³C18-linoleic acid (*n* = 3). Data are presented as mean ± SEM and statistical significance was assessed by two-tailed unpaired Student’s *t*-test. **p* < 0.05 vs CON group.

**Fig.S6. Elovl7 knockdown alleviated lipid metabolism disorder and LC-PUFA synthesis**

1. Representative immunoblots of Elovl7 in si-NC or si-Elovl7 cells.
2. Quantification of Elovl7 blots normalized to β-actin in si-NC or si-Elovl7 cells (*n* = 3). Data are presented as mean ± SEM and statistical significance was assessed by one-way ANOVA followed by Tukey post-hoc test. **p* < 0.05 vs si-NC group; ns, not significant for any comparison.
3. OPLS-DA plots displaying repertoires of targeted lipidomics from CON or ADR-treated wide-type and si-Elovl7 cells. Each point represents a sample (*n* = 3). Ellipses represent 95% confidence regions.
4. Heatmap showing relative contents of oxidative PLs in wide-type or si-Elovl7 cells with or without ADR treatment.
5. Heatmap showing relative contents of PI and PA with tails of different length and saturation in wide-type or si-Elovl7 cells with or without ADR treatment.
6. mRNA expression of genes related to ferroptosis (*Alox15*, *Lpcat3*, *Gpx4*, *Slc7a11*), fatty acid desaturation (*Fads1*), triacylglycerol synthesis (*Dgat2*, *Plin2*) and de novo synthesis (*Fasn*) (*n* = 5). Data are presented as mean ± SEM and statistical significance was assessed by one-way ANOVA followed by Tukey post-hoc test. ***p* < 0.01, ****p* < 0.001, *****p* < 0.0001 vs si-NC group; *^#^p* < 0.05, *^##^p* < 0.01, *^####^p* < 0.0001 vs “si-NC+ADR” group; ns, not significant.
7. Quantification of Acsl4 expression in podocytes by immunofluorescence co-localization (*n* = 3).
8. Quantification of Elovl7 expression in podocytes by immunofluorescence co-localization (*n* = 3). Data are presented as mean ± SEM and statistical significance was assessed by one-way ANOVA followed by Tukey post-hoc test. **p* < 0.05 vs CTL group; ns, not significant.
